# An ALS-Linked Mutant SOD1 Produces a Locomotor Defect Associated with Aggregation and Synaptic Dysfunction When Expressed in Neurons of *Caenorhabditis elegans*

**DOI:** 10.1371/journal.pgen.1000350

**Published:** 2009-01-23

**Authors:** Jiou Wang, George W. Farr, David H. Hall, Fei Li, Krystyna Furtak, Lars Dreier, Arthur L. Horwich

**Affiliations:** 1 Howard Hughes Medical Institute, Yale School of Medicine, New Haven, Connecticut, United States of America; 2 Department of Genetics, Yale School of Medicine, New Haven, Connecticut, United States of America; 3 Department of Neuroscience, Albert Einstein College of Medicine, Bronx, New York, United States of America; 4 Department of Molecular, Cellular, and Developmental Biology, University of Michigan, Ann Arbor, Michigan, United States of America; 5 Department of Neurobiology, David Geffen School of Medicine, University of California Los Angeles, Los Angeles, California, United States of America; The Jackson Laboratory, United States of America

## Abstract

The nature of toxic effects exerted on neurons by misfolded proteins, occurring in a number of neurodegenerative diseases, is poorly understood. One approach to this problem is to measure effects when such proteins are expressed in heterologous neurons. We report on effects of an ALS-associated, misfolding-prone mutant human SOD1, G85R, when expressed in the neurons of *Caenorhabditis elegans*. Stable mutant transgenic animals, but not wild-type human SOD1 transgenics, exhibited a strong locomotor defect associated with the presence, specifically in mutant animals, of both soluble oligomers and insoluble aggregates of G85R protein. A whole-genome RNAi screen identified chaperones and other components whose deficiency increased aggregation and further diminished locomotion. The nature of the locomotor defect was investigated. Mutant animals were resistant to paralysis by the cholinesterase inhibitor aldicarb, while exhibiting normal sensitivity to the cholinergic agonist levamisole and normal muscle morphology. When fluorescently labeled presynaptic components were examined in the dorsal nerve cord, decreased numbers of puncta corresponding to neuromuscular junctions were observed in mutant animals and brightness was also diminished. At the EM level, mutant animals exhibited a reduced number of synaptic vesicles. Neurotoxicity in this system thus appears to be mediated by misfolded SOD1 and is exerted on synaptic vesicle biogenesis and/or trafficking.

## Author Summary

A new animal model of the human neurodegenerative disease amyotrophic lateral sclerosis (ALS; Lou Gehrig's Disease) is presented. Two percent of ALS cases result from heritable mutations affecting the abundant enzyme superoxide dismutase (SOD1). Such mutations have been indicated to impair the folding and stability of the enzyme, leading it to misfold and aggregate in motor neurons, associated with the paralyzing disease. Here, when a mutant form of human SOD1 was produced in neurons of *C. elegans* worms, it led to a severe locomotor defect—the worms were essentially paralyzed. The protein formed aggregates in the neurons, including an intermediate form of aggregate, soluble oligomers, that has been linked to toxicity to cells. By contrast, worms expressing the normal version of human SOD1 protein exhibited normal movement and no aggregation. The movement defect was further analyzed using chemical inhibitors and found to result from defective function of synapses, the connections made between neurons, and between neurons and muscle. Finally, in a screen using RNA interference, we observed that the worms' aggregation and locomotor condition was worsened when a class of molecules called molecular chaperones, which assist protein folding in the cell, were impaired in function. This is consistent with the idea that misfolded SOD1 is directly involved with causing the neuronal dysfunction.

## Introduction

A number of neurodegenerative diseases have been associated with protein misfolding and aggregation, with a specific protein in each case observed to aggregate in a particular population of neurons. For example, in the case of amyotrophic lateral sclerosis (Lou Gehrig's Disease), a dominantly inherited form of this condition, accounting for ∼2% of cases, is associated with mutant forms of the abundant cytosolic homodimeric enzyme superoxide dismutase (SOD1), which accumulate in insoluble aggregates in motor neurons [1]–[3]. Mutational studies of SOD1-linked ALS have uncovered single residue substitutions throughout the enzyme subunit [4], and studies in vitro indicate that the substitutions generally destabilize the protein, disposing to misfolding and aggregation [5]–[7]. It remains unknown, however, exactly how apparent misfolding and aggregation of SOD1 exerts toxic effects on motor neurons. Is there a central common effect shared by the various mutant alleles that comprises a common pathway of motor neuron injury?

Mice transgenic for a variety of mutant SOD1 alleles also develop motor neuron disease resembling that of affected humans [8],[9], enabling a variety of pathological and biochemical studies. A survey of pathology reported for various alleles implicates a variety of potential physical sites of toxicity, including mitochondria, endoplasmic reticulum, and axonal traffic. For example, abnormal-appearing mitochondria have been observed in animals transgenic for G93A and G37R SOD1 [9]–[11], and several mutant SOD1's have been coisolated with spinal cord mitochondria [12]–[15]. Concerning ER function, an unfolded protein response (UPR) was observed in spinal cord of G93A mice [16], and a recent report suggests that mutant SOD1 induces this response by binding to the ER membrane component Derlin-1, blocking retrograde traffic of ER proteins to the cytosol for proteasomal degradation (ERAD) at the level of ubiquitination [17]. Concerning axonal traffic, both anterograde and retrograde transport have been observed to be retarded in mice transgenic for mutant SOD1 [18]–[24]. Which, if any, of these effects is primary to mutant SOD1-induced motor neuron damage?

One approach to resolving this question is to produce mutant human SOD1 in neurons in an invertebrate system to inspect for effects on function, with the idea that this might reveal a minimal target of the toxic effect which could ultimately be further evaluated in the mammalian system. Such an approach has been taken, for example, with other neurodegenerative disease-associated proteins, expressing them in *Drosophila* or *C. elegans*, including polyglutamine repeat proteins [e.g. 25],[26] and α-synuclein [e.g. 27],[28]. Here we have taken such an approach with SOD1 using *C. elegans*, programming pan-neuronal expression of a mutant version of human SOD1, G85R, that obligatorily misfolds. We observe a locomotor defect in transgenic animals expressing the mutant SOD1, associated with aggregation of the mutant SOD1 protein and with synaptic dysfunction, involving deficient numbers and possibly deficient trafficking of pre-synaptic vesicles.

## Results

### Pan-Neuronal Expression of an ALS-Associated Mutant Version of Human SOD1 in *C. elegans* Produces Locomotor Defects Associated with Intra-Neuronal Aggregation

To examine the effects of an ALS-associated human mutant SOD1 on a collective of neurons in an optically accessible nervous system, we produced transgenic *C. elegans* expressing G85R mutant or wild-type human SOD1 (referred to hereafter as SOD). G85R SOD has been identified in human cases of ALS [1], and G85R SOD transgenic mice develop a similar disease [29]. In the latter setting, the protein has been shown to behave as a misfolded monomer [30]. That is, it fails to form the normal SOD homodimer, and it lacks the normal disulfide bond that is formed between Cys 57 and Cys 146 when the protein is properly folded. (This disulfide bond is normally formed despite localization of SOD to the relatively reducing cytosol). To express the wild-type and G85R SOD proteins in as many of the 302 neurons of a *C. elegans* hermaphrodite as possible, a pan-neuronal promoter, the promoter of the *C. elegans* synptobrevin gene (*snb-1*), was used to drive the respective wild-type and G85R human cDNAs encoding SOD. To allow direct observation of the expressed SOD proteins, two additional constructs were employed that join a YFP reporter sequence via a flexible peptide linker to the C-terminus of the wild-type or mutant SOD [31]. Multiple stable transgenic *C. elegans* lines of both unfused and fused constructs were produced.

### Locomotor Defects

**Figure 1:**
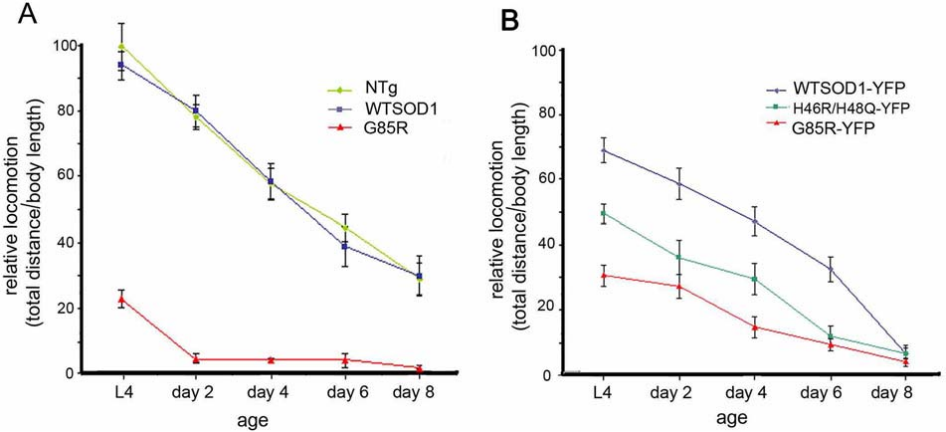
Locomotor defects in G85R and G85R-YFP transgenic *C. elegans*. Rates of forward movement were measured as net distance traveled during 30 sec, divided by body length, normalized against non-transgenic L4 animals. The assay was carried out immediately after transfer to a fresh bacterial plate and measured by videomicroscopy. N = 20 for each time point; error bars = SEM. A, unfused constructs. B, YFP fusions. Transgenic lines employed were WTSOD (line 7), G85R (line 10), WTSOD-YFP (line 51), G85R-YFP (line 18) and H46R/H48Q-YFP (line 7).

We noticed immediately that G85R SOD transgenic animals exhibited minimal forward movement across the culture medium compared with normal movement of age and protein expression-matched wild-type SOD transgenic strains (Figure 1A and Video S1; see Figure S1 for immunoblot analysis and activity blot analysis). The wild-type SOD transgenic animals exhibited a forward movement speed similar to that of the parental nontransgenic *C. elegans* N2 strain (Figure 1A). In addition to severely reduced forward crawling of the mutant transgenic animals, their side-to-side thrashing movement in liquid was also severely affected (Figure S2 and Videos S2, S3). The rates of forward movement of the animals transgenic for the fusion proteins, WTSOD-YFP and G85R-YFP, likewise showed a large difference (Figure 1B), although the mutant now exhibited significant forward movement, and the wild-type fusion was somewhat slower than the nonfused wild-type transgenic animals (compare Figure 1B with 1A). These same effects were observed on thrashing (Figure S2). Thus the addition of the YFP moiety has effects on the mutant and wild-type SOD proteins, exerted in opposite directions. Overall, however, even with the YFP fusion protein, there was still markedly slower movement of the G85R animals as compared with the wild-type (Figure 1B).

To assess whether the biological behavior of the G85R-YFP fusion protein has any relevance to the mammalian context where expression is associated with an identifiable clinical neuronal disorder, G85R-YFP was produced in transgenic mice from a human SOD genomic clone with YFP fused to the last coding exon. This fusion produced an ALS phenotype in mice at 3–9 months of age, with the age of onset of the motor deficit depending on copy number and expression level of the transgene (JW, GF, KF, and ALH, unpublished). By contrast, expression-matched wild-type SOD-YFP transgenic mice produced in parallel did not develop disease even at ages of 2 years and beyond. Thus the SOD-YFP fusions tested here reflect the behavior of the nonfused counterparts in the context of production of ALS-like disease in the mammalian setting.

An additional SOD mutant transgenic *C. elegans* strain, H46R/H48Q-YFP, containing an SOD double mutant allele that blocks copper binding by SOD and produces ALS in transgenic mice [32], was also examined. Here it produced a movement defect less prominent than that seen in G85R-YFP (Figure 1B).

### Aggregation in Neurons of G85R-YFP Transgenic Animals

**Figure 2:**
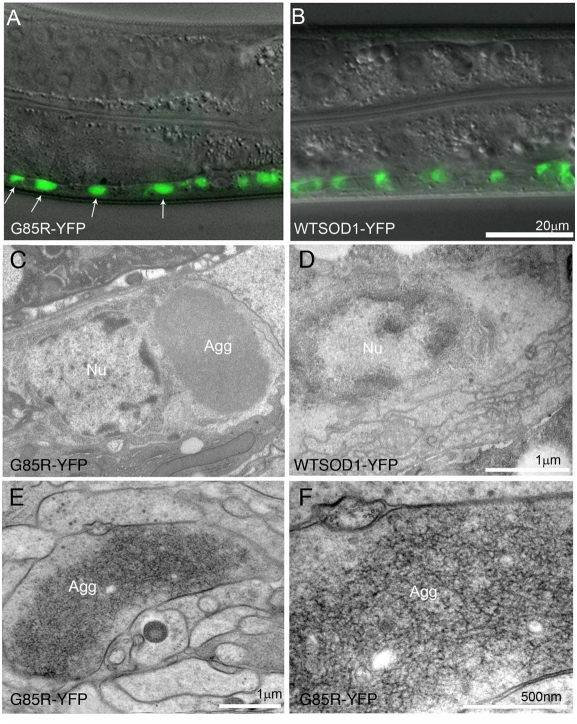
Aggregation in G85R and G85R-YFP neurons. A, B, Fluorescence analyses of G85R-YFP and WTSOD-YFP transgenic animals at stage L4, comparing cytoplasmic fluorescence in cell bodies of ventral nerve cord. WTSOD-YFP (panel B) exhibits a diffuse pattern in cell bodies (with noticeable nuclear exclusion) while G85R-YFP (panel A) exhibits a more discrete pattern in brighter, well defined, zones. C–F, EM analyses. Aggregate (Agg) in the perinuclear region of a ventral cord cell body of a G85R-YFP day 4 adult animal (panel C), and normal appearance of cell body of a WTSOD-YFP day 4 adult (panel D); Nu: nucleus. G85R (nonfused) shows fibrillar-appearing aggregate in a large nerve ring process (panels E,F). Panels C, D from chemical immersion fixed preparation and E, F from high pressure freezing preparation.

Fluorescence microscopy of transgenic *C. elegans* at both larval and adult stages revealed fluorescence in many neurons in the nerve ring (head region), the ventral nerve cord, lateral body wall, and tail ganglia (Figure S3A). Non-neuronal fluorescence of the spermatheca and two gonadal distal tip cells was also observed. The character of fluorescence of neuronal cell bodies of G85R-YFP transgenic animals differed from that of WTSOD-YFP transgenics as exemplified by cell bodies of motor neurons along the ventral nerve cord (Figure 2A, B). [Neuronal processes, by contrast, did not exhibit altered fluorescence (Figure S3B).] Cell bodies from the wild-type animals exhibited a more diffuse cytosolic fluorescence pattern, whereas those from the G85R-YFP mutant exhibited a well-demarcated pattern, suggestive of aggregate formation. Consistent with this, a FRAP experiment (fluorescent recovery after photobleaching) showed very slow recovery of fluorescence in mutant cell bodies as compared with wild-type (see Figure S4).

Aggregates were directly observed in the mutant by EM examination following chemical fixation (Figure 2C, D), which revealed well-delineated electron-dense inclusions in the cytosol of ventral nerve cord cell bodies of G85R-YFP animals (Figure 2C, Agg.). By contrast, there were no recognizable aggregates in WTSOD-YFP cell bodies (Figure 2D). The location of G85R-YFP aggregates in a perinuclear position is reminiscent of aggresomes [33] or of perinuclear structures distinct from the centrosome known as JUNQ, involved in juxtranuclear quality control [34], but aggregates formed in the unfused G85R animals exhibited a more diffuse, “fluffy” character (Figure S5A, asterisks). When high pressure freezing/fixation was employed on the G85R animals, these regions were now observed as amorphous inclusions (e.g. Figure 2E, Agg.), which at high magnification appeared to contain loosely stacked fibrillar material (Figure 2F).

#### Biochemical fractionation

**Figure 3:**
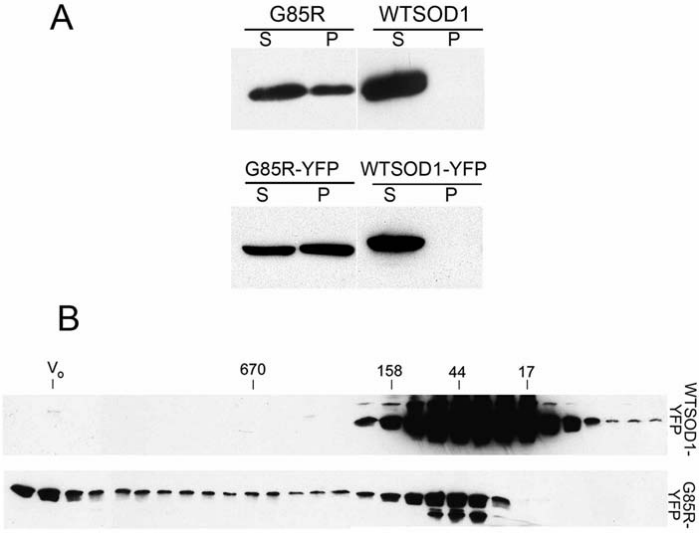
Biochemical analysis of SOD solubility and assembly state in transgenic animals. A, Fractionation of worm extracts into soluble and insoluble fractions under native conditions and Western blotting. Extract was prepared by sonication and cuticle debris removed, followed by centrifugation at 120,000×g×15 min to produce soluble (S) and insoluble pellet (P) fractions. G85R and G85R-YFP exhibit substantial insoluble material whereas none is detected in wild-type. B, Western blot analysis of fractions from gel filtration chromatography of soluble fraction, showing soluble G85R-YFP oligomers extending from monomer-size up to the void volume. WTSOD-YFP, by contrast, shows only lower molecular weight species. Numbers above the blot panels indicate the size in kDa of standard proteins chromatographed on the same column; V_o_ = void volume, corresponding to ∼5 MDa size.

Biochemical analysis also revealed insoluble SOD protein as well as soluble oligomers to be present specifically in mutant animals. Animals were homogenized in non-denaturing buffer, the extracts were centrifuged at 120,000×g×15 min, and the supernatant and pellet fractions were solubilized under reducing conditions and analyzed by immunoblotting with anti-SOD antibodies. As shown in Figure 3A, both G85R and G85R-YFP animals exhibited a substantial portion of SOD protein in the insoluble fraction (P) whereas all of the SOD protein in wild-type animals localized to the soluble fraction (S). The soluble fractions of G85R-YFP and WTSOD-YFP were further analyzed by gel filtration chromatography (under reducing conditions), and immunoblotting of the fractions revealed the presence of soluble oligomers specifically present in the mutant extending in size from ∼45 kDa (size of the fusion monomer) up to ∼5 MDa (void volume). Such soluble oligomers are likely to be the precursors to insoluble aggregates and have been implicated in cellular toxicity [35]–[37].

#### Distribution

**Figure 4:**
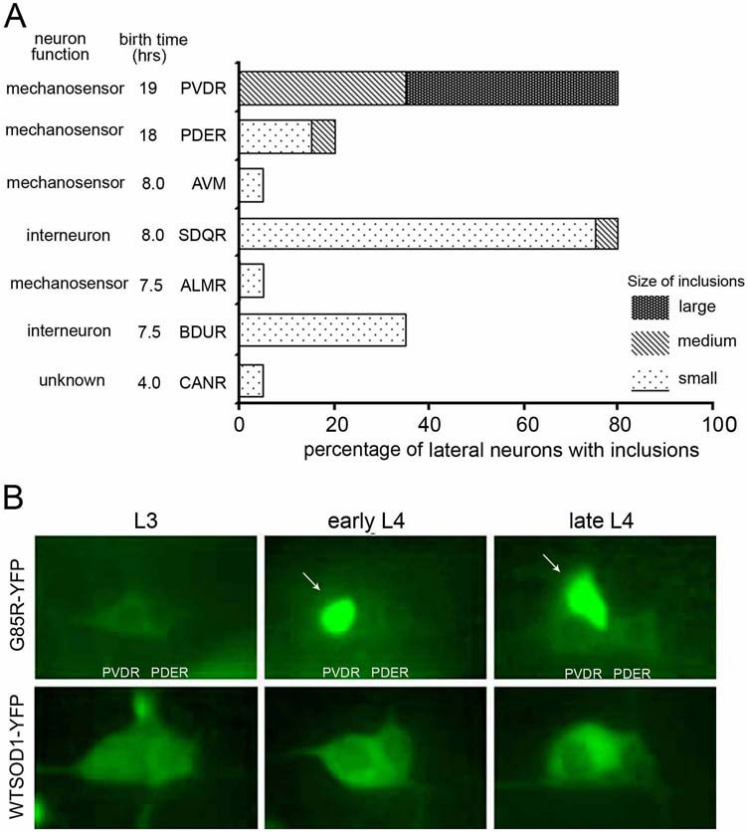
Distinct neuronal pattern of aggregation in G85R-YFP animals. A, Involvement of selected lateral neurons. Mid-L4 G85R-YFP animals were scored; N = 21. Note that neuronal function and birth time do not appear to correlate with aggregate formation. B, PVDR generally presents with a well demarcated fluorescent inclusion whereas PDER, lying next to it, is usually unaffected in G85R-YFP animals. Arrows point to cytosolic aggregates.

To address the distribution of neurons containing aggregates, first we counted fluorescent aggregates in the ventral nerve cord cell bodies (all belonging to motor neurons) at stage L4. Individual cell bodies could not be readily assigned due to their high density, but we observed greater than 40 aggregate-containing cell bodies out of the total of 56 cell bodies of the ventral nerve cord. The aggregates in these cell bodies were typically of large size, like those shown in Figure 2A. Next, a number of individually identifiable neurons of the lateral body wall were scored in L4 stage G85R-YFP animals for presence or absence of a visible fluorescent aggregate (Figure 4A). This revealed that some neurons, e.g. PVDR and SDQR, nearly always contained an aggregate, whereas other neurons only rarely exhibited an aggregate. Of interest in regard to such differences is the behavior of the two neighboring lateral body wall mechanosensory neurons, PVDR and PDER (Figure 4B). PVDR nearly always exhibited fluorescent aggregates, usually first observed at the L4 stage, whereas PDER was generally spared (Figure 4B; note that PDER was present in G85R-YFP animals, delineated by diffuse green fluorescence not clearly visible in this image). The nature of the difference between these two neurons that governs the different aggregation behavior is interesting to contemplate and may relate to specific neuronal activity. More generally, whether neurons which have sequestered misfolded SOD-YFP into an insoluble fraction are functionally more affected in vivo than neurons lacking aggregates, as suggested by Matsumoto et al [31], who observed reduced viability of SOD-YFP aggregate-containing cultured cells, or, conversely, are protected by aggregation, as suggested by primary neuron studies with Htt^ex1^-polyQ-GFP [38], is unknown.

Concerning temporal development of aggregates, fluorescent aggregates in the head region and ventral cord were detectable as early as L1, while lateral body wall aggregates were not detected until L3. Aggregation was not restricted to motor neurons, as indicated in Figure 4. Concerning other sensory neurons, we note that the DiI-stainable chemosensory amphid neurons in the anterior region, as well as the stainable phasmid tail neurons, exhibited normal DiI staining, but no green fluorescence was observed in these cells in either mutant or wild-type animals, leaving uncertain whether any expression of SOD-YFP occurred in these neurons. Consistent with broad neuronal involvement, however, additional general functions of mutant animals were found to be affected, including survival, brood size, and rate of development (see Figures S6, S7).

### Abnormal Processes and Synaptic Function in G85R Animals

#### Ventral nerve cord processes are abnormal

**Figure 5:**
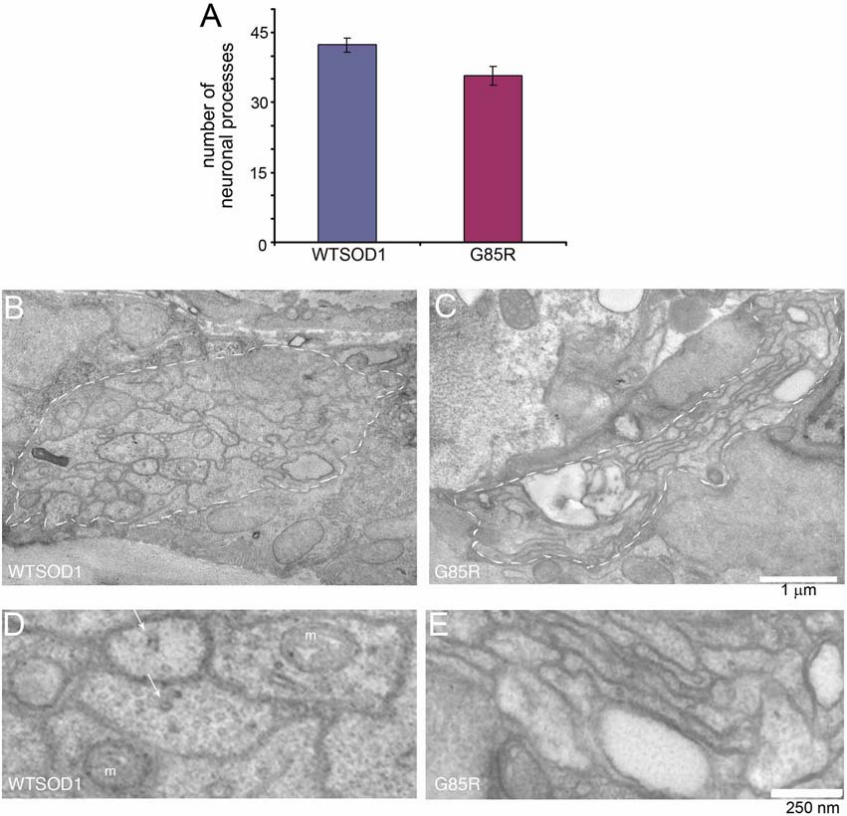
Ventral nerve cord is affected in 4 day old adult G85R animals – fewer and smaller diameter processes and lack of organelles. A, Number of neuronal processes is mildly reduced. Transverse sections of transgenic animals were prepared and examined by EM, and the number of neuronal processes in the main bundle of the ventral nerve cord was determined. N = 9. (Error bars indicate SEM.) B–E, Representative cross-sections of WTSOD and G85R animals at two levels of magnification, with white dashed lines in B, C denoting the boundaries of the main bundle of processes. The diameter of processes was reduced, and the number of organelles including mitochondria (m) and synaptic vesicles (arrows) was greatly reduced in G85R.

In further EM studies, transverse sections of the ventral nerve cord were prepared and the processes examined. In 4 day old adults, the number of processes (mostly axons) in G85R animals was slightly reduced compared with wild-type (Figure 5A). More striking, however, were morphologic abnormalities that included reduced diameter (compare Figure 5B, C), and reduced numbers of organelles, both mitochondria and vesicles, within them (compare Figure 5D, E). Some of the processes in G85R animals were also swollen by large vesicular structures (Figure 5C). In contrast with these abnormalities of the ventral nerve cord, the innervated body wall muscles appeared normal, with normal sarcomeres and subcellular organelles (not shown).

#### Synaptic vesicles are reduced in dorsal region

**Figure 6:**
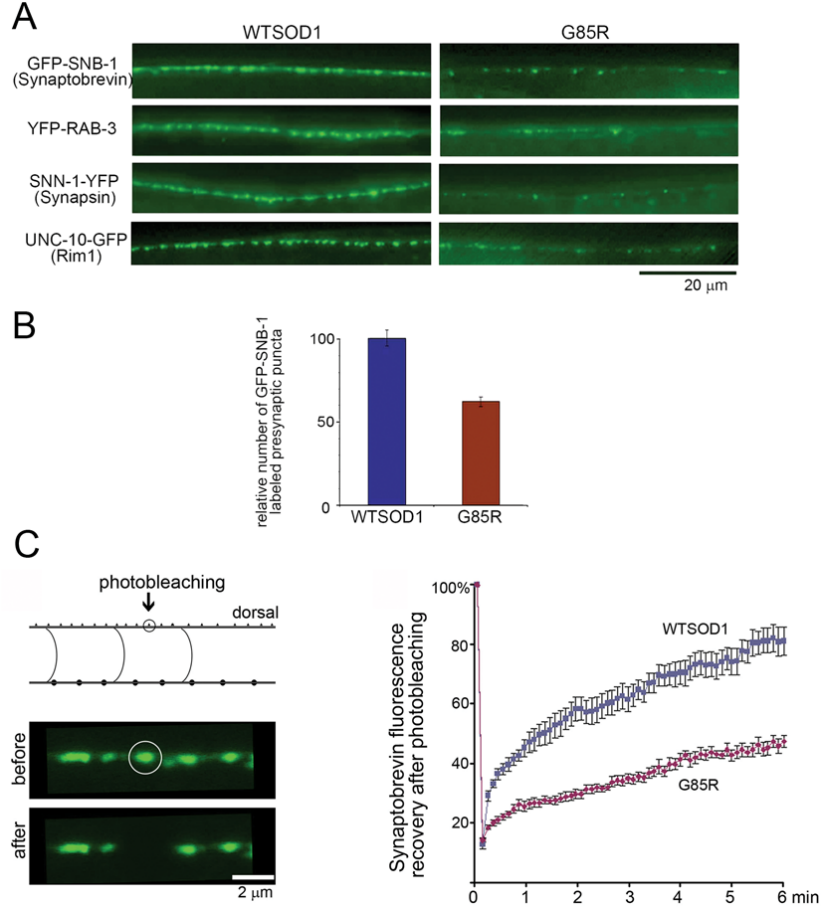
Dorsal nerve cord of G85R at L4 stage exhibits diminished numbers and brightness of puncta of fluorescent presynaptic markers and diminished fluorescence recovery of GFP-synaptobrevin after photobleaching. A, Three different fluorescent protein-tagged synaptic proteins, synaptobrevin (SNB-1), RAB-3, and synapsin-1 (SNN-1), and one tagged presynaptic active zone protein, Rim1, were examined in WTSOD and G85R transgenic worms. Anterior portion of the dorsal cord is shown. In all cases there was a reduced number of puncta in the mutant animals. This was quantitated in panel B for GFP-SNB-1 animals, examining 10 of each genotype at L4 stage. Puncta were counted along a 50 micron distance and normalized to total body length. The number was reduced in the mutant animals, p = 0.0004. Error bars = SEM. Panel C, fluorescent recovery after photobleaching (FRAP) of GFP-synpatobrevin. Left, example of photobleaching of dorsal cord. Right, recovery of fluorescence plotted as function of time. Mid-L4 animals were subject to photobleaching, covering an area of 1 µm surrounding the dorsal punctum of interest, and fluorescence was recorded thereafter. Average intensity was normalized to the pre-bleaching intensity. G85R showed significantly slower recovery. N = 8 for each genotype. Error bars are SEM.

To address whether synapses might be affected, labeling was carried out with GFP-synaptobrevin. This protein, when expressed in DA motor neurons, has been observed to produce a continuous pattern of fluorescent puncta in the dorsal nerve cord, where neuromuscular junctions are present (e.g. ref. 39). Wild-type and G85R (unfused) transgenic animals were crossed to P*unc-129*::GFP-synaptobrevin transgenic animals, and the double transgenic progeny were examined for the number of dorsally located green synaptic puncta that demarcate neuromuscular junctions (Figure 6A). Whereas wild-type animals exhibited a continuous band of such puncta, the mutant animals exhibited a discontinuous pattern. In addition to the reduced number of puncta (Figure 6B), their signals were also reduced. A similar decrease in number and brightness of puncta relative to wild-type was also observed when the fluorescent-tagged synaptic vesicle proteins YFP-RAB-3 and synapsin-1-YFP (SNN-1-YFP) were similarly crossed in and examined (Figure 6A). These data suggest that both synapse number and vesicle content in the dorsal cord is reduced. Consistent with decreased numbers of synapses, when the presynaptic active zone protein UNC-10 (RIM1) [40] was examined, it exhibited a diminished number of puncta in the mutant (Figure 6A).

A FRAP experiment was also carried out on the GFP-synaptobrevin animals, showing slower recovery of fluorescence at puncta in the dorsal region in G85R animals vs wild-type (Figure 6C). This potentially reflects abnormal dynamic behavior of synaptic vesicles in this dorsal region.

#### EM analysis shows a diminished number of presynaptic vesicles

To inspect synapses at the ultrastructural level, EM studies of the nerve ring were carried out, because this region has the greatest concentration of synapses. Thin sections were prepared from 4 day old adult G85R or wild-type animals using the high pressure freezing method. The character of synapses appeared to be affected (Figure S8). For example, in many synapses of the mutant animals (panels C,D), there appeared to be a paucity of synaptic vesicles which was more pronounced in the region closest to the presynaptic density (37 of 50 synapses examined) as compared with wild-type (panels A,B), where vesicles were densely packed in the presynaptic region including the active zone and periactive zone (44 of 50 synapses examined). In view of the paucity of vesicles, further EM analysis was carried out to identify whether there might be a corresponding accumulation of vesicles in cell bodies, as occurs for example in *unc-104* kinesin-deficient animals [41], but this was not observed. Instead, vesicles were observed in some cases to be trapped within aggregate material.

#### Deficient synaptic function measured by aldicarb assay

**Figure 7:**
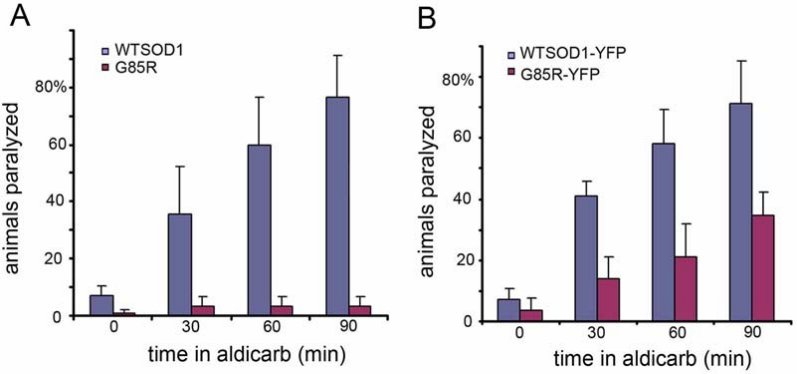
Aldicarb paralysis assay of SOD transgenic strains. G85R and G85R-YFP transgenic animals of L4 stage are relatively resistant to aldicarb. A, Comparison of WTSOD and G85R transgenic animals at various times after exposure to aldicarb, measuring percentage that fail to exhibit movement upon physical prodding in the head region. B, Comparison of WTSOD-YFP and G85R-YFP animals for percent exhibiting aldicarb paralysis. Error bars are SEM.

To assess synaptic function, the effects of the cholinesterase inhibitor alidcarb in paralyzing wild-type or G85R transgenic animals was measured [39],[42]. Resistance to this inhibitor can be caused by loss of cholinergic synaptic transmission. Both G85R and G85R-YFP animals were strongly resistant to aldicarb as compared with the corresponding wild-type animals (Figure 7), indicating deficient synaptic function. To distinguish whether postsynaptic function was contributing, a second compound, levamisole, a cholinergic receptor agonist, was employed. G85R animals displayed the same sensitivity to levamisole-induced paralysis as wild-type (data not shown). Thus both morphologic and functional assays point to defective presynaptic function as a proximate cause to the locomotor defect of G85R transgenic animals.

### RNAi Screen for Modifiers of Protein Aggregation—Components that Increase Aggregation

To identify genetic modifiers of neuronal aggregation in the G85R SOD-YFP transgenic animals, we carried out an RNAi screen. Neurons of *C. elegans* are relatively resistant to RNA interference, so alleles previously identified to enhance interference activity, *eri-1* (*mg366*) and *lin-15B* (*n744*), were introduced [39]. Strikingly, the introduction of these alleles led to a significant reduction in fluorescence from the G85R-YFP protein as compared with the parental strain (Figure S9A), associated with a decrease in the number of fluorescent inclusions observed in the ventral nerve cord (Figure S9B) and with somewhat improved movement. These results are consistent with a previous report that *lin-15B* could produce silencing of multicopy transgenes [43]. We observed that either allele alone could produce substantial silencing of the G85R-YFP transgene, although *lin-15B* exerted a greater effect. The combination of alleles proved to sufficiently reduce fluorescence and aggregate formation of G85R-YFP to a degree that could allow for a “dynamic” range of effects of RNAi screening, i.e. both reduction and enhancement of green fluorescence patterns reflecting aggregation would be detectable. A bacterial feeding library was employed for RNAi testing. Collectives of animals at all stages were transferred onto feeding plates and visually examined by multiple observers after 3–6 days for changes in the number and intensity of fluorescent aggregates in the nerve ring and ventral nerve cord as compared with the strain fed bacteria with an empty vector.

In the general case of observing decreased fluorescent aggregates when animals were placed on a particular interfering bacterial strain, the development of the animals was generally also slowed and viability in many cases reduced, reflecting likely effects on general health or on general gene expression, and these interfering RNAs were not studied further. There was one exception to this, involving an interfering RNA for a ubiquitin specific protease, where fluorescent aggregation was reduced and viability somewhat improved, and this gene is under further study.

In the general case of animals with increased fluorescent aggregates on a particular interfering bacterial strain, these animals generally exhibited larger numbers of fluorescent inclusions and diminished locomotion. There were 88 such hits (Table S1). 7 of these appeared likely to suppress *eri-1*; *lin-15B* action and were not studied further (see last entries in Table S1), while the remaining 81 hits were categorized into 10 groups (Table 1). The largest group of well-annotated hits comprised molecular chaperones and quality control components, amounting to about a quarter of the hits. These are discussed below. The other large group, of about the same size, comprised a collective of uncharacterized gene products.

**Table 1:** Summary of 81 RNA interference hits that worsened aggregation.

Protein chaperones, turnover, and modification	22	27.2%
Redox	3	3.7%
Signal transduction	7	8.6%
Transcription, RNA processing	6	7.4%
Metabolism	6	7.4%
DNA replication and repair	4	4.9%
Extracellular matrix	2	2.5%
Translation	2	2.5%
Intracellular trafficking	2	2.5%
Uncategorized	27	33.3%

To independently confirm RNAi hits in the parental genetic background, loss-of-function alleles were obtained for 12 hits of interest and were crossed with the parental G85R-YFP strain. 11 out of 12 of these enhanced the inclusion phenotype of G85R-YFP. For example, heat shock factor 1 (HSF1), which transcriptionally regulates a number of stress components [44], registered very strongly in the RNAi screen in increasing aggregate formation. Consistently, when the *sy441* allele of *hsf-1* was crossed into G85R-YFP, a strong increase in aggregate formation was observed (Figure S10A), and locomotion of these animals was substantially decreased as compared with either parental strain (Figure S10B).

Multiple interference hits of strong magnitude were observed in a number of pathways (Table 2), indicating that these pathways are likely playing a role in preventing aggregation of the misfolded G85R-YFP protein or facilitating its turnover. For example, hits of a collective of chaperone components registered strong increases of aggregation, including an Hsp110 (*C30C11.4*), a DnaJ (A2) (*dnj-19*), an Hsp70 (*stc-1*), and a neuron specific Hsp16 (*F08H9.4*). Three of these were validated as strongly increasing aggregation when mutant alleles were crossed with G85R-YFP. Two hits were identified in the ubiquitin-mediated turnover pathway at the level of E3 ligase SCF complexes, SEL-10, an F-box protein, and RBX-1, a RING finger protein, the latter confirmed with the *ok782* knockout allele. Three hits were also observed in the sumoylation pathway by the RNAi screen, *uba-2*, encoding the E1 enzyme that activates SUMO, *ubc-9*, which encodes one subunit of the E2 SUMO-conjugating heterodimer, and an E3 SUMO-ligase component *gei-17* (homologous to PIAS1). In validation of the interference effects, an allele of SUMO, *smo-1* (*OK359*), increased aggregation when crossed into the parental G85R-YFP strain. The latter hits raised the question of whether G85R-YFP is itself sumoylated, and this is under study. In addition, three components involved with redox regulation were identified, PDI-2, an orthologue of a human thioredoxin domain-containing protein (*C30H7.2*), and BLI-3, a dual oxidase with both a peroxidase domain and a superoxide-generating oxidase domain.

**Table 2:** Selected genes whose inactivation strongly aggravates formation of SOD-YFP neuronal inclusions^1^.

CATEGORY	GENE	FUNCTION	2RNAI SCORE	3ALLELE SCORE
*Chaperone/quality control*	*hsf-1* (Y53C10A.12)	Heat shock transcription factor	5	*sy441* ++
	*C30C11.4*	homolog to human apg-1 (a heat shock 110 kDa protein)	3	*gk533* ++
	*dnj-19 (T05C3.5)*	homolog to DnaJ subfamily A member 2	3	*gk649* ++
	*F08H9.4*	neuron-specific HSP16	3	*ok1976* ++
	*stc-1 (F54C9.2)*	member of HSP70 superfamily (microsome associated)	3	
*Protein turnover*	*sel-10 (F55B12.3)*	member of the CDC4/CUL-1 family of ubiquitin ligases	3	
	*rbx-1 (ZK287.5)*	RING box protein RBX1, a subunit of the SCF ubiquitin-ligase complex	2	ok782 +
	*W07G4.4*	Predicted aminopeptidase	4	
*Protein modification*	*uba-2 (W02A11.4)*	sumo activating enzyme	5	
	*ubc-9 (F29B9.6)*	sumo conjugating enzyme	4	
	*gei-17 (W10D5.3)*	Homologous to E3 SUMO-protein ligase PIAS1	3	
*Redox*	*bli-3 (F56C11.1)*	dual oxidase	3	*e767* ++
	*pdi-2 (C07A12.4)*	Protein disulfide isomerase	2	*gk375* ++
	*C30H7.2*	thioredoxin domain-containing protein precursor	2	
*Signal transduction*	*dbl-1 (T25F10.2)*	member of the TGFβ superfamily	3	*nk3* +
*Dopamine metabolism*	*dat-1 (T23G5.5)*	plasma membrane dopamine transporter	3	*tm903* +
*Dna replication & repair*	*top-1 (M01E5.5)*	DNA topoisomerase I	5	
	*div-1 (R01H10.1)*	homolog of the B subunit of the DNA polymerase alpha-primase complex	3	*or148* ++
*Transcription*	*H43I07.2*	RNA polymerase I and III, subunit RPA40/PRC40	3	
*Longevity factor*	*pha-4 (F38A6.1)*	FoxA transcription factor	4	

1 20 genes out of the 88 hits from the screen are listed here.

2 RNAi scores range from 1 to 5, with 5 representing the strongest increase of the G85R-YFP inclusions, and 1 a discernible increase.

3 Alleles are loss-of-function mutations that have been bred to homozygosity in the strain carrying the G85R-YFP transgene; “+” represents moderate increase of the inclusion profile, and “++” strong increase.

Other pathways were also identified through strong effects of interference. For example, a hit in the TGFβ component DBL-1 was confirmed by the allele *nk3*. DBL-1 is expressed in neuronal cells, e.g. in the ventral cord, has recently been implicated in GABAergic synaptic transmission [45], and has been shown to affect both body size and male tail development [46]. Notably, in mammalian contexts, TGF-β family members have exhibited effects on axon outgrowth and protection from excitotoxicity [e.g. 47]. A hit of the dopamine transporter DAT-1 was confirmed with allele *tm903*. A link between neuronal activity and aggregation in the cases of GABAergic and dopaminergic transmission thus seems a consideration but requires further study. In the DNA replication/repair pathways, interference with *top-1*, the gene for topoisomerase 1, had a very strong effect, equivalent to the strong effect of *hsf-1* interference. How such action could figure into aggregation behavior remains unclear. Similarly, interference of *div-1*, a subunit of the DNA polymerase α/primase complex, increased aggregation. Also a strong effect was observed with interference of *pha-4*, a FoxA transcription factor, implicated in calorie-restriction-mediated longevity, at least in part by regulating endogenous *C. elegans* SOD enzymes [48].

## Discussion

### Neuronal Presynaptic Dysfunction in G85R SOD Transgenic Worms

We have described here that pan-neuronal expression of a human ALS-associated mutant SOD in *C. elegans* produces substantial locomotor defects associated with macroscopic aggregation in neuronal cell bodies. By contrast, a wild-type human SOD produced neither locomotor defects nor aggregation. Further testing indicated that the human SOD mutant, G85R, unable to fold properly and producing both soluble oligomers and aggregates, appears to produce neuronal dysfunction in *C. elegans* at the presynaptic level. This was indicated by three types of observation. First, there was a reduced number and brightness of puncta of fluorescently-labeled synaptobrevin, RAB-3, and synapsin, as well as of the presynaptic active zone protein RIM1 in the dorsal nerve cord in transgenic G85R animals as compared with wild-type SOD transgenic animals. Second, mutant transgenic animals but not wild-type exhibited resistance to aldicarb paralysis, consistent with deficient acetylcholine release at cholinergic synapses in the mutant. Third, EM studies on a small number of animals revealed paucity of vesicles in the presynaptic region of nerve ring synapses of the mutant animals. In addition, EM showed reduced numbers of vesicles and mitochondria in ventral nerve cord processes (axons) of transgenic mutant animals. In contrast with the foregoing findings pointing to presynaptic dysfunction, there was a lack of postsynaptic effects. The cholinergic agonist levamisole produced the same efficient paralysis of both mutant G85R and wild-type SOD transgenic animals. In addition, EM inspection of muscle revealed normal morphology.

The putative presynaptic defect could result, in the first instance, from defective biogenesis, axonal transport, or recycling of synaptic vesicles. The diminution of vesicles in dorsal and ventral nerve cord processes and their sparsity in the presynaptic regions as demonstrated from the EM and fluorescence studies suggest that biogenesis or transport is affected. Because accumulations of vesicles were not detected in cell bodies, it seems less likely that transport is affected. Yet the failure of recovery of the GFP-synaptobrevin fluorescence following photobleaching could be consistent with an ongoing transport or recycling defect. Further studies will be required to resolve the steps affected.

Excitingly, effects of expression of human SOD1 on synaptic transmission in another invertebrate system have recently been reported. Watson et al [49] observed that expression of human SOD1 (wild-type or mutant) in motor neurons of *Drosophila* produced an age-progressive climbing defect that was associated in electrophysiological studies, stimulating muscle through the giant fiber circuit, with progressive loss of muscle response during high frequency stimulation.

Presynaptic effects have also been observed in several contexts in *C. elegans* expressing a number of other neurodegeneration-associated proteins. Animals transgenic for both wild-type and FTDP-associated mutant forms of tau presented with locomotor defects, associated with aldicarb resistance (and levamisole sensitivity), followed by appearance of macroscopic aggregates and then apparent neuronal loss [50]. Animals transgenic for α-synuclein exhibited locomotor defects when any of a number of synaptic proteins were knocked down, associated with aldicarb resistance and levamisole sensitivity [28]. In contrast with these studies, however, the studies of SOD1 presented here indicate a direct and specific effect of the mutant G85R SOD1 on presynaptic function.

### Misfolding and Aggregate Formation and Linkage to Locomotor Defect

So far it is not evident how or whether the presence of G85R-SOD aggregates in cell bodies of specific neurons, comprising a variety of neuronal cell types in the transgenic mutant animals, directly relates to the apparent presynaptic defect. We note, however, that the degree of locomotor defect correlates roughly with the degree of aggregation observed. For example, for the G85R-YFP transgene, out of multiple stable integrant lines, those with the highest level of fluorescence and greatest level of aggregation exhibited the strongest locomotor defect. Thus, either the aggregates themselves or perhaps the apparent precursors, soluble oligomers (Figure 3B), may be directly responsible for the presynaptic defect. The defect could come at the level of physical interactions of oligomers or aggregates either directly with synaptic vesicles themselves or with soluble or cytoskeletal components that are involved with vesicle biogenesis and traffic. A recent study producing pan-neuronal expression of dimeric versions of human wild-type and G85R SOD1 in *C. elegans* also observed locomotor defects associated with aggregation, but, in contrast to the direct correlation above, observed that a heterodimeric G85R-WTSOD-GFP molecule, while producing less aggregation than G85R-G85R-GFP, led to a greater paraquat-induced reduction of animal survival [51]. Thus in the context of a heterodimer, residual SOD enzymatic activity may contribute to toxicity.

### Lack of Neuronal Cell Death

Notably, despite evident protein aggregation and synaptic dysfunction, neurons in the mutant animals studied here were not subject to cell death, even during later adult life. This resembles the observation in the recent report of Watson et al [49] where human SOD1 expressed in *Drosophila* motor neurons produced both focal SOD protein accumulation and measurable electrical dysfunction but no observable cell death. Similarly, in earlier studies of *C. elegans* PLM neurons transgenic for polyglutamine expansion, touch sensitivity function was abolished but cell death did not occur [52]. This lack of cell death may either relate to the state of disease progression or be a function of the invertebrate neuronal systems themselves. For example, concerning stage of disease, in the mammalian context, SOD1-affected motor neurons appear likely to be functionally affected for a period of time before being subject to cell death. In *C. elegans* or *Drosophila*, by contrast, the trajectory may not extend sufficiently in time to produce cell death, albeit that added insults such as oxidative toxicity [50] may be capable of producing cell death. Alternatively, these systems may differ from that of mammals. *C. elegans* has, for example, a limited number of glia, and they may not function as in the mammalian context to hasten death of affected neurons [53]–[55]. Alternatively, the absence of cell death could be a function of a different neuronal response to chronic exposure to misfolded protein as compared with mammalian neurons.

### Proteostatic Protection from Misfolded SOD in *C. elegans* Neurons

The RNA interference screen conducted here, examining relative levels of G85R-YFP protein aggregation in relation to knockdown of various gene products, validated in many cases by crosses with corresponding mutant alleles (Table 2), provided evidence that a proteostatic network similar to that present in body wall muscles of *C. elegans* [56],[57] and elsewhere [44] is operative in *C. elegans* neurons. Whether it is induced in response to mutant human SOD1 remains to be determined, and whether a higher level of expression of some or all members of the network could be protective remains to be tested. Two transcriptional regulators that lie at the top of such a network were identified, HSF-1 and PHA-4/FoxA, which have a broad range of targets in, for example, chaperone pathways [44] and redox regulation [48], respectively. Components within the network were also identified, including chaperones, an E3 ligase, and redox components. Additional components had effects on aggregation but their mechanism of action remains unclear, including SUMO, the TGF-β homologue, DBL-1, expressed mainly in neurons, and two components of DNA maintenance, topoisomerase I and a subunit of polα/primase complex, implicating DNA integrity in the response. Notably absent from both the interference screen and from EM studies was evidence for involvement of the autophagy system. Consistent with this, administration of rapamycin was without effect on the extent of aggregation (data not shown).

### Relation of the Worm G85R SOD-Induced Phenotype to G85R SOD-Associated ALS in Mouse

Do the phenotypic properties observed here bear any relation to mammalian disease induced by expression of mutant SOD1? Could the transgenic *C. elegans* inform usefully about mammalian disease? As mentioned, the G85RSOD-YFP fusion protein expressed in mice indeed produces an ALS-like disease whereas WTSOD-YFP fusion produces no ill effect. Interestingly, the presynaptic effects observed here with the G85R transgenic *C. elegans* may have a parallel in mutant G85R SOD transgenic mice, as reported recently by Caroni and colleagues [58], who examined motor neuron axons and NMJs in hind limb muscle of mutant transgenic animals of varying age [see also 59]. In vulnerable motor neuron axons (FF and FR), they observed at early time (7 months of age; 2 months before end-stage) localized synaptic vesicle accumulation associated with diminished overall density of vesicles, reflecting apparent stalling of vesicle traffic, followed at a later stage by severe loss of synaptic vesicles associated with loss of presynaptic active zone markers. The findings at later time generally agree with the observations here in dorsal cord of our G85R *C. elegans*, where both fluorescent synaptic vesicle protein markers and an active zone marker (RIM1) appear to be reduced (Figure 6). The nature of the vesicle trafficking defect in either setting remains to be elucidated. Does it reflect failure of vesicles to be produced in the first instance, as suggested by the lack of organelles in ventral cord processes here (Figure 5), and/or is it a block of recycling, as might be suggested by the FRAP analysis of GFP-synaptobrevin (Figure 6C)? Is it a direct effect of mutant SOD, forming physical association with synaptic vesicles? Or is it a secondary effect, mediated e.g. via the motor/cytoskeletal trafficking system? Questions concerning both the basis to vesicular defects and the overall pathway of toxicity of mutant SOD protein remain to be resolved.

## Materials and Methods

### DNA

The *C. elegans snb-1* promoter, a PCR-amplified genomic DNA segment extending from minus 3021 bp to just upstream of the SNB start codon, was inserted in place of the *unc-54* enhancer/promoter in the plasmid pPD30_38 (Fire Lab Vector Kit, Addgene Inc., Cambridge, MA), and the various human SOD cDNA-containing segments were then adjoined. SOD mutations were generated by PCR, and the derived coding sequences confirmed by sequencing them in entirety. Fusion constructs joining human SOD with YFP via a linker segment (LQLQASAV) were kindly provided by Dr. R. Morimoto, Northwestern University [31].

### *C. elegans* Strains and Methods

The N2 Bristol strain of *C. elegans* was used as the wild-type strain. Standard culturing and genetic methods were used [60]. Animals were maintained at 20°C unless otherwise indicated. Mutant strains obtained from the Caenorhabditis Genetics Center (CGC), the National Bioresource Project in Japan, and the lab of Joshua Kaplan are listed in Suppl. Table 2. Germline transformation was performed by injecting DNA solution containing 20 ng/µl of an SOD construct and 5 ng/µl of *myo2::GFP* into hermaphrodite gonads [61]. Multiple extrachromosomal lines were established based on the fluorescent markers. They were further treated with trimethylpsoralen/UV to generate integrated lines that stably expressed the transgenes. At least three independent stable lines were produced for each variant, and each line was backcrossed with the N2 strain four times. The transgenic SOD and SOD-YFP lines used in these studies are designated in the legend to Figure 1. *nuIs152* has the transgene *Punc-129::GFP-snb-1* [39]. *nuIs168* has the transgene *Punc-129::YFP-Rab-3* [62]. *nuIs163* and *nuIs165* strains containing *Punc-129::snn-1-YFP* and *Punc-129::unc-10-GFP* were the kind gift of Joshua Kaplan [63].

### Microscopy

For high resolution imaging, animals were immobilized with levamisole and examined by either differential interference contrast (DIC) or fluorescence with an Olympus IX81 microscope equipped with spinning disk confocal illumination. For fluorescence recovery after photobleaching (FRAP), a laser confocal microscope was used (Zeiss LSM510 META).

For transmission electron microscopy, animals were prepared by conventional two-step chemical immersion fixation or high-pressure-freezing [64],[65]. Serial thin sections were prepared and post-stained with heavy metals. At least four animals of each genotype were analyzed using a Tecnai 12 Biotwin at 80 kV.

### Locomotion Analysis

A video-based assay was used to assess the locomotion speed of *C. elegans*. Animals were transferred to a plate with a fresh bacterial lawn on which movement tracks could be traced. Immediately upon release, worms exhibit a maximum movement response for a short duration. A 30 second movie was shot for each worm, and the ratio of the movement distance to the body length, measured by the NIH ImageJ software, was used as a movement index (see Video S1).

### Aldicarb Sensitivity Assay

Mid L4 animals were transferred to freshly made NGM agar plates without bacterial food containing 1 mM aldicarb, and at different time points the animals were prodded on the nose to determine whether they had reached complete paralysis [42]. An identical experiment with 1 mM levamisole was also performed.

### Protein Analysis

To assess solubility of SOD protein, animals were disrupted by sonication on ice in 0.5 ml extraction buffer (PBS, 1 mM EDTA, 1 mM EGTA, 1 mM TCEP, with half a tablet of Complete Mini protease inhibitor cocktail (Roche)) and left on ice for 10 min to allow large debris, including cuticle, to sediment. The supernatant fraction was then centrifuged in a Beckman TLA-100 rotor at 53,000 rpm (>120,000×g) for 15 min at 4°C. Pellets were washed once by resuspension in the extraction buffer and sedimentation. Supernatant and SDS-solubilized pellet fractions were analyzed in SDS-PAGE under reducing conditions. Supernatant fractions (1 mg total protein) were also subjected to gel filtration chromatography on a Superose 6 gel filtration column (GE Healthcare) and eluted with PBS supplemented to 0.1 mM TCEP at 0.5 ml/min. Individual fractions (0.5 ml) were examined by SDS-PAGE and Western blotting. For Western analysis, antibodies to human SOD1 (SOD-100, Stressgen, Canada), antibodies raised in rabbits against purified YFP (Cocalico), or antibodies to actin (C4, MP Biomedicals, Inc., Aurora, Ohio) were used.

### RNAi Feeding Screen and Further Tests with Loss-of-Function Alleles

An RNAi feeding library of 16,757 bacterial clones was employed for screening (GeneService, Cambridge, UK). Animals at mixed ages were screened in 96-well plates at 15°C as described [66]. “Hits” were identified by an increased number and intensity of fluorescent neuronal inclusions using a Leica fluorescence stereoscope with a 2.0× PLANAPO lens. All positives were subjected to secondary screening at both 15°C and 20°C in 6-well plates. The identities of all positive RNAi clones were confirmed by DNA sequencing of the plasmid insert. For selected hits, where loss-of-function alleles were available, the corresponding strains were crossed to the G85R-YFP parental strain (line 8). The genotypes of the product strains were verified by PCR or PCR/DNA sequencing and the phenotypes studied.

## Supporting Information

Figure S1 Amount and activity of human SOD expressed from a pan-neuronal promoter in transgenic *C. elegans* strains. A, Top, Immunoblot probed with anti-human SOD1 antibody (no cross-reaction with *C. elegans* SOD); Bottom, identical amounts of sample probed with anti-actin antibody in blot of gel identical to that in top panel. NTg, non-transgenic Bristol N2 strain of *C. elegans*. 10 µg of total protein was applied for each lane. Transgenic lines were WTSOD1 (line 23), G85R (line 10), WTSOD1-YFP (line 51), H46R/H48Q-YFP (line 7), and G85R-YFP (line 18). Note that G85R routinely migrates faster than WTSOD. This effect is also present, although less apparent, for the fusion proteins. B, Top, In-gel activity assay [67] carried out on native gel in which soluble extract (120,000×g×15 min) of worm strains had been fractionated. 100 µg protein was applied to each lane. Activity of wild-type human SOD1 (and its fusion), but not that of the mutant SOD1's was observed (no activity of endogenous *C. elegans* SOD was detected). Bottom, Western blot of denaturing gel loaded with one-tenth the amount of sample as that in top panel, probed with anti-human SOD1 antibody. In both cases, only the relevant section of the gel or blot is shown.
(0.69 MB TIF)

Figure S2 Thrashing rate of transgenic *C. elegans* strains. L4 animals were transferred to a drop of M9 buffer at 20°C and after 1 min of adaptation the number of body bends was counted for 1 min. N = 37. Error bars are SEM.
(0.27 MB TIF)

Figure S3 Expression pattern of Psnb1::human SOD1-YFP in *C. elegans* assessed by fluorescence imaging. A, Lateral view of stably-transformed WTSOD1-YFP transgenic L4 animal showing expression of fluorescent protein in nerve ring region, in ventral nerve cord, in dorsal nerve cord, as well as in lateral neuronal cell bodies, and in tail region. Arrow indicates position at which lateral wall neuron shown in panel B is situated. B, Lateral view of ALM neuron in lateral wall of L4 animals. Head of worm is to left. Arrows point to processes and arrowhead points to aggregate in cell body of the G85R-YFP neuron.
(0.88 MB TIF)

Figure S4 Fluorescence recovery after photobleaching (FRAP) of ventral nerve cord cell bodies of G85R-YFP and WTSOD1-YFP transgenic *C. elegans*. Black circle denotes location of photobleaching. n, nucleus.
(1.09 MB TIF)

Figure S5 Diffuse aggregate formation in the cytosol of cell bodies of ventral nerve cord of G85R transgenic animals but not in WTSOD1 transgenics. Altered appearance of cytosol (asterisks), with “fluffy” character and with no discernible organelles in this section of a neuron cell body of a G85R animal (left), distinct from normal cytosol in cell body of a WTSOD1 transgenic animal (right). Note that the fluffy inclusion has pushed the nucleus away from the center of the cell body, similar to the position of the dense aggregate in Figure 2C. Day 4 adults were prepared by chemical immersion fixation. Scale bar 1 µm.
(1.77 MB TIF)

Figure S6 Survival curves and brood sizes of transgenic animals. A, B Survival of animals was followed from mid-L4, with death determined by failure to respond to mechanical prodding. N = 45 for each genotype. C, D Brood size was determined by counting eggs laid during the lifetime of hermaphrodite. Lines used were WTSOD1 (line 23), G85R (line 10), WTSOD1-YFP (line 51), and G85R-YFP (line 18). N = 24 for each genotype. Error bars are SEM. All animals were cultured under standard conditions at 20°C. The presence of G85R mutation affects these parameters, with a more severe effect of the G85R-YFP fusion.
(0.63 MB TIF)

Figure S7 Rate of larval development of transgenic animals. Individual animals at L1 stage were selected and scored for developmental progression after 40 hr and 60 hr. G85R-YFP fusion animals were strongly delayed in development compared with G85R. N = 30 for each genotype; error bars = SEM.
(0.76 MB TIF)

Figure S8 Paucity of pre-synaptic vesicles in G85R transgenic animals. Representative transverse sections of pharyngeal nerve ring from day 4 adult animals prepared by high-pressure-freezing method, with white arrows pointing to presynaptic density from the postsynaptic side. Note the paucity of presynaptic vesicles overall, and stronger depletion close to the presynaptic density in the G85R animals (panels C,D) compared with robust numbers of presynaptic vesicles in WTSOD animals (panels A,B). Scale bar, 500 nm.
(1.39 MB TIF)

Figure S9 The double mutant background *eri1;lin-15B* that facilitates RNA interference in neurons reduces the expression/fluorescence of G85R-YFP, with corresponding reduction of the number of fluorescent inclusions. A, G85R-YFP transgenic animals of different stages with or without presence of *eri-1*(*mg366*); *lin-15B*(*n744*) were imaged. L1 and L2, whole animal views. L4 and adult, ventral cord region is shown. Bright puncta along ventral cord correspond to fluorescent inclusions in cell bodies. B, numbers of fluorescent inclusions in ventral nerve cord on successive days in the same animals. N = 18 for each genotype; error bars = SEM.
(0.73 MB TIF)

Figure S10 Effects of several mutant alleles on aggregation and locomotion of G85R-YFP animals. A, The *hsf-1* loss-of-function allele *sy441* causes an increase of aggregation. Nerve ring and anterior ventral cord of L1 stage animals are shown. B, *sy441* and mutant alleles of *dbl-1* and *div-1* produce defective forward movement in L4 stage G85R-YFP animals. N2 locomotion (not shown) was set to 100%.
(0.84 MB TIF)

Table S1 RNAi screening hits.
(0.10 MB XLS)

Table S2 Strains.
(0.06 MB DOC)

Video S1 Crawling movement defects in *C. elegans* expressing mutant SOD1 in neurons. A representative G85R-YFP hermaphrodite and a WTSOD-YFP control were transferred to a fresh plate with OP50 E. coli, and their movements were video-recorded. Animals are day 2 adults.
(11.14 MB MOV)

Video S2 Thrashing movement. Thrashing movement defects in *C. elegans* expressing mutant SOD1 in neurons. A representative G85R hermaphrodite (S2) and a WTSOD control (S3) were transferred to M9 buffer, and their thrashing was video-recorded. Animals are at the L4 stage.
(2.87 MB MOV)

Video S3 Wild-type thrashing. Thrashing movement defects in *C. elegans* expressing mutant SOD1 in neurons. A representative G85R hermaphrodite (S2) and a WTSOD control (S3) were transferred to M9 buffer, and their thrashing was video-recorded. Animals are at the L4 stage.
(2.99 MB MOV)
