# Supplementary figures and images for: An ALS-Linked Mutant SOD1 Produces a Locomotor Defect Associated with Aggregation and Synaptic Dysfunction When Expressed in Neurons of Caenorhabditis elegans
Source: PLoS Genet. 2009 Jan 23;5(1):e1000350. doi: 10.1371/journal.pgen.1000350 (PMC2621352; doi:10.1371/journal.pgen.1000350)

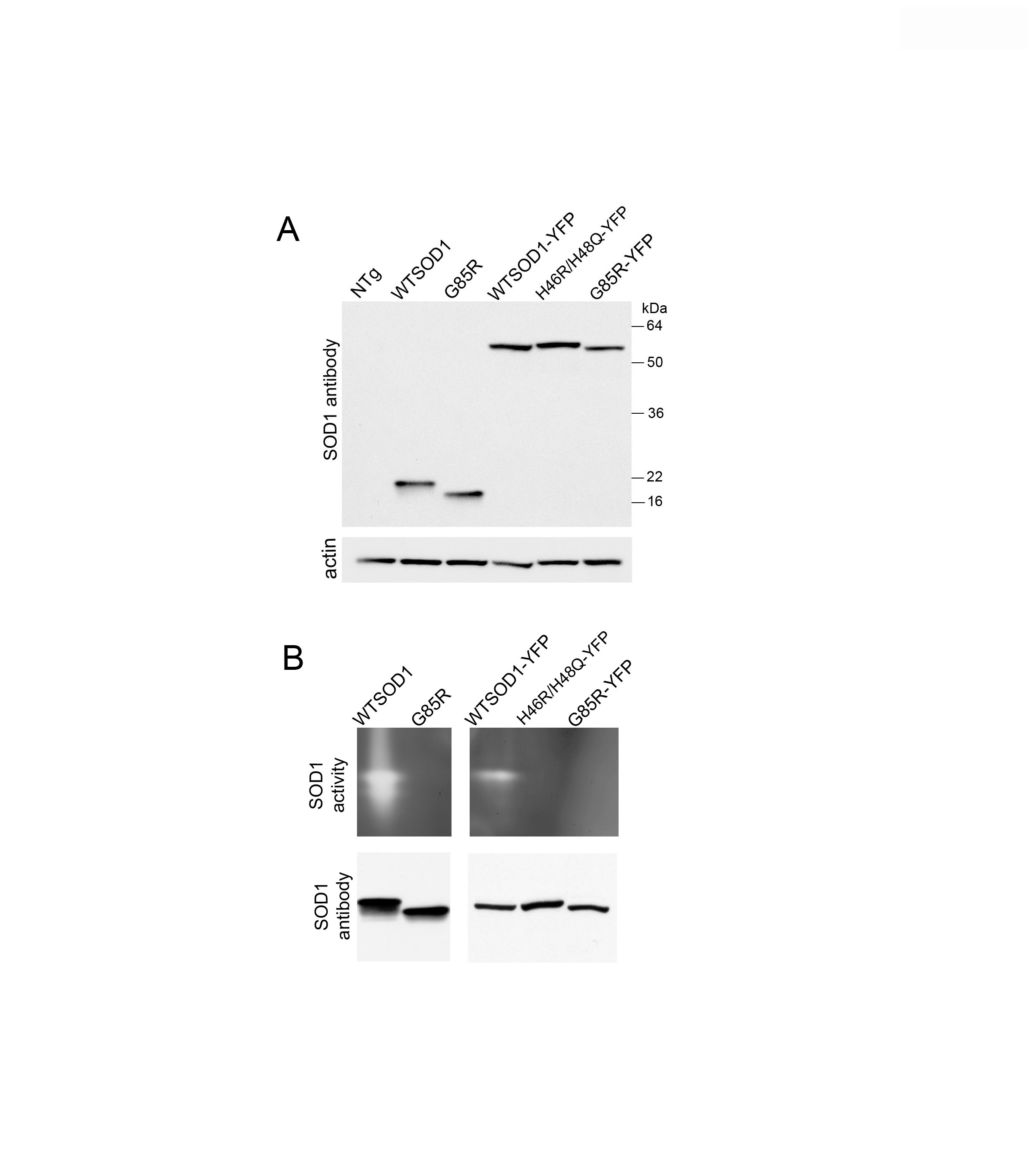

Supplement: Figure S1 — Amount and activity of human SOD expressed from a pan-neuronal promoter in transgenic C. elegans strains. A, Top, Immunoblot probed with anti-human SOD1 antibody (no cross-reaction with C. elegans SOD); Bottom, identical amounts of sample probed with anti-actin antibody in blot of gel identical to that in top panel. NTg, non-transgenic Bristol N2 strain of C. elegans. 10 µg of total protein was applied for each lane. Transgenic lines were WTSOD1 (line 23), G85R (line 10), WTSOD1-YFP (line 51), H46R/H48Q-YFP (line 7), and G85R-YFP (line 18). Note that G85R routinely migrates faster than WTSOD. This effect is also present, although less apparent, for the fusion proteins. B, Top, In-gel activity assay [67] carried out on native gel in which soluble extract (120,000×g×15 min) of worm strains had been fractionated. 100 µg protein was applied to each lane. Activity of wild-type human SOD1 (and its fusion), but not that of the mutant SOD1's was observed (no activity of endogenous C. elegans SOD was detected). Bottom, Western blot of denaturing gel loaded with one-tenth the amount of sample as that in top panel, probed with anti-human SOD1 antibody. In both cases, only the relevant section of the gel or blot is shown. (0.69 MB TIF) [file pgen.1000350.s001.tif]

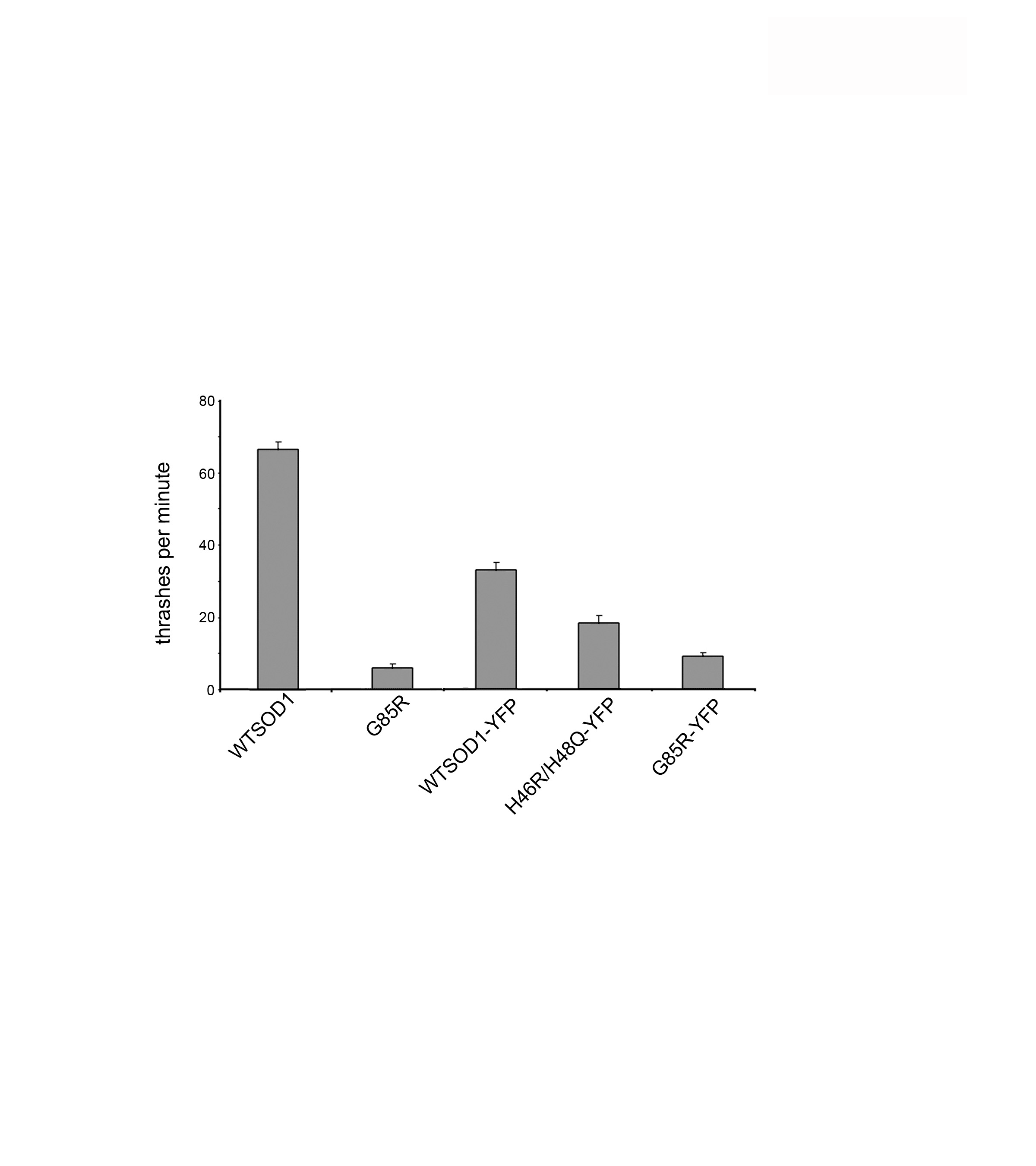

Supplement: Figure S2 — Thrashing rate of transgenic C. elegans strains. L4 animals were transferred to a drop of M9 buffer at 20°C and after 1 min of adaptation the number of body bends was counted for 1 min. N = 37. Error bars are SEM. (0.27 MB TIF) [file pgen.1000350.s002.tif]

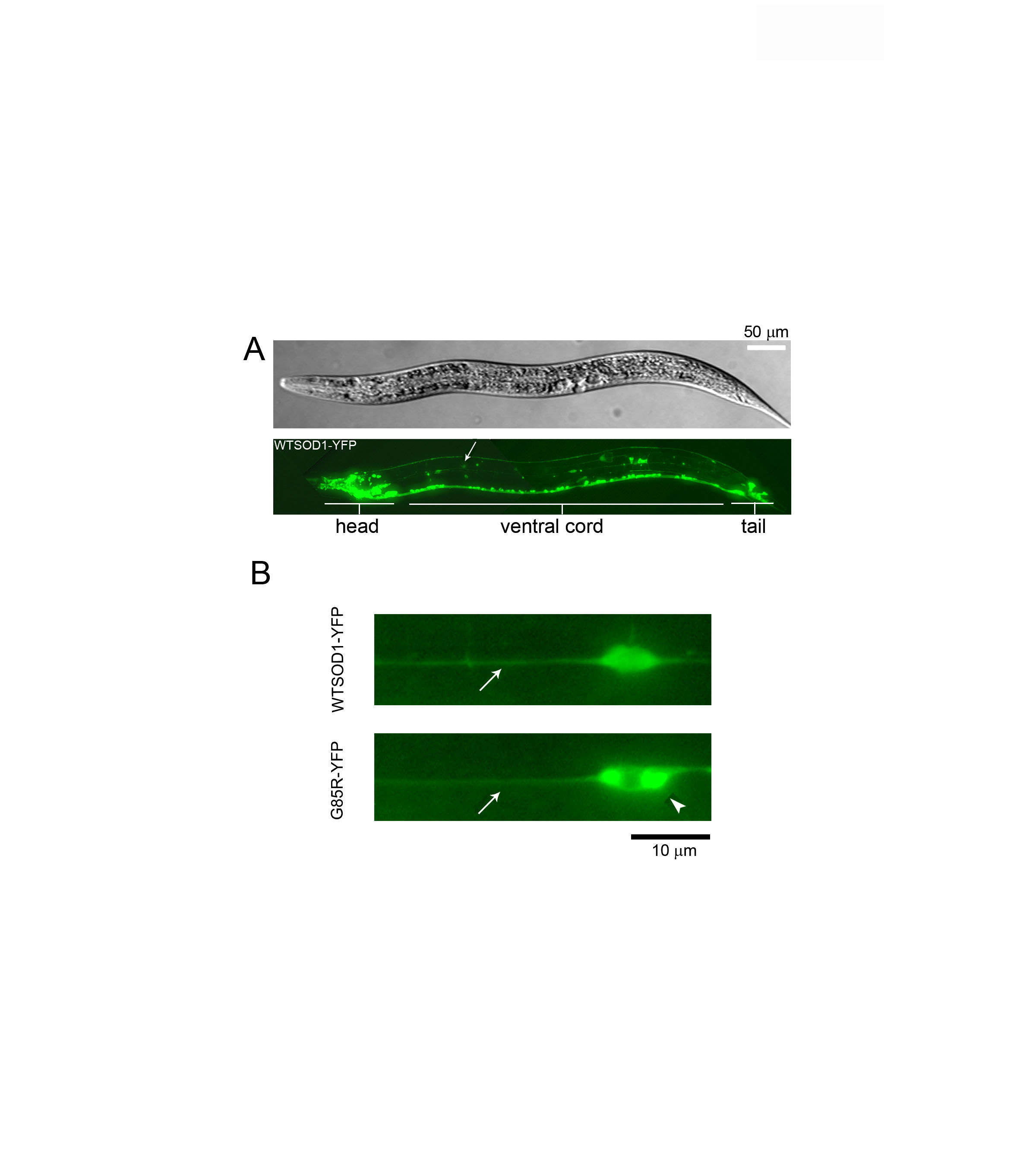

Supplement: Figure S3 — Expression pattern of Psnb1::human SOD1-YFP in C. elegans assessed by fluorescence imaging. A, Lateral view of stably-transformed WTSOD1-YFP transgenic L4 animal showing expression of fluorescent protein in nerve ring region, in ventral nerve cord, in dorsal nerve cord, as well as in lateral neuronal cell bodies, and in tail region. Arrow indicates position at which lateral wall neuron shown in panel B is situated. B, Lateral view of ALM neuron in lateral wall of L4 animals. Head of worm is to left. Arrows point to processes and arrowhead points to aggregate in cell body of the G85R-YFP neuron. (0.88 MB TIF) [file pgen.1000350.s003.tif]

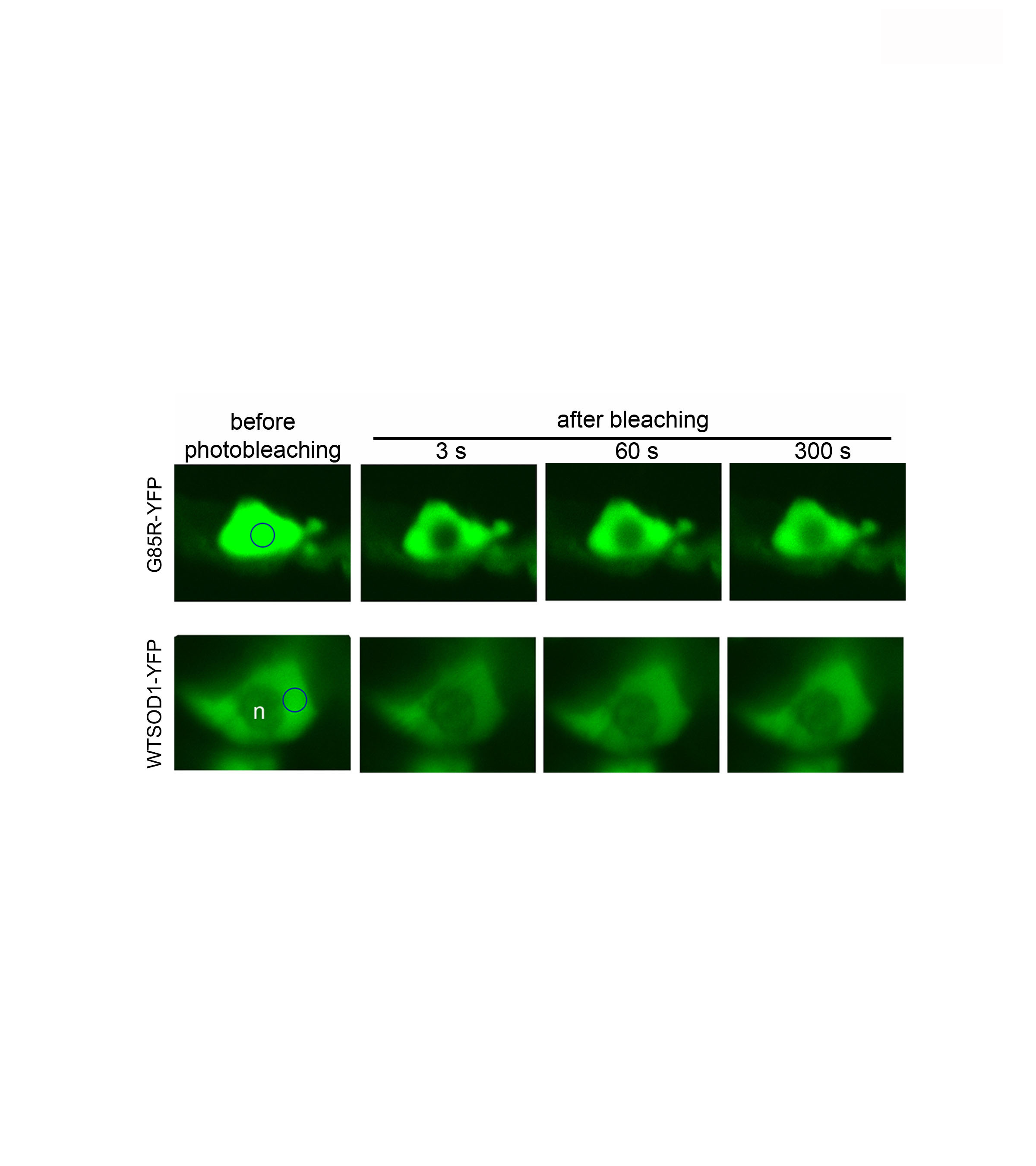

Supplement: Figure S4 — Fluorescence recovery after photobleaching (FRAP) of ventral nerve cord cell bodies of G85R-YFP and WTSOD1-YFP transgenic C. elegans. Black circle denotes location of photobleaching. n, nucleus. (1.09 MB TIF) [file pgen.1000350.s004.tif]

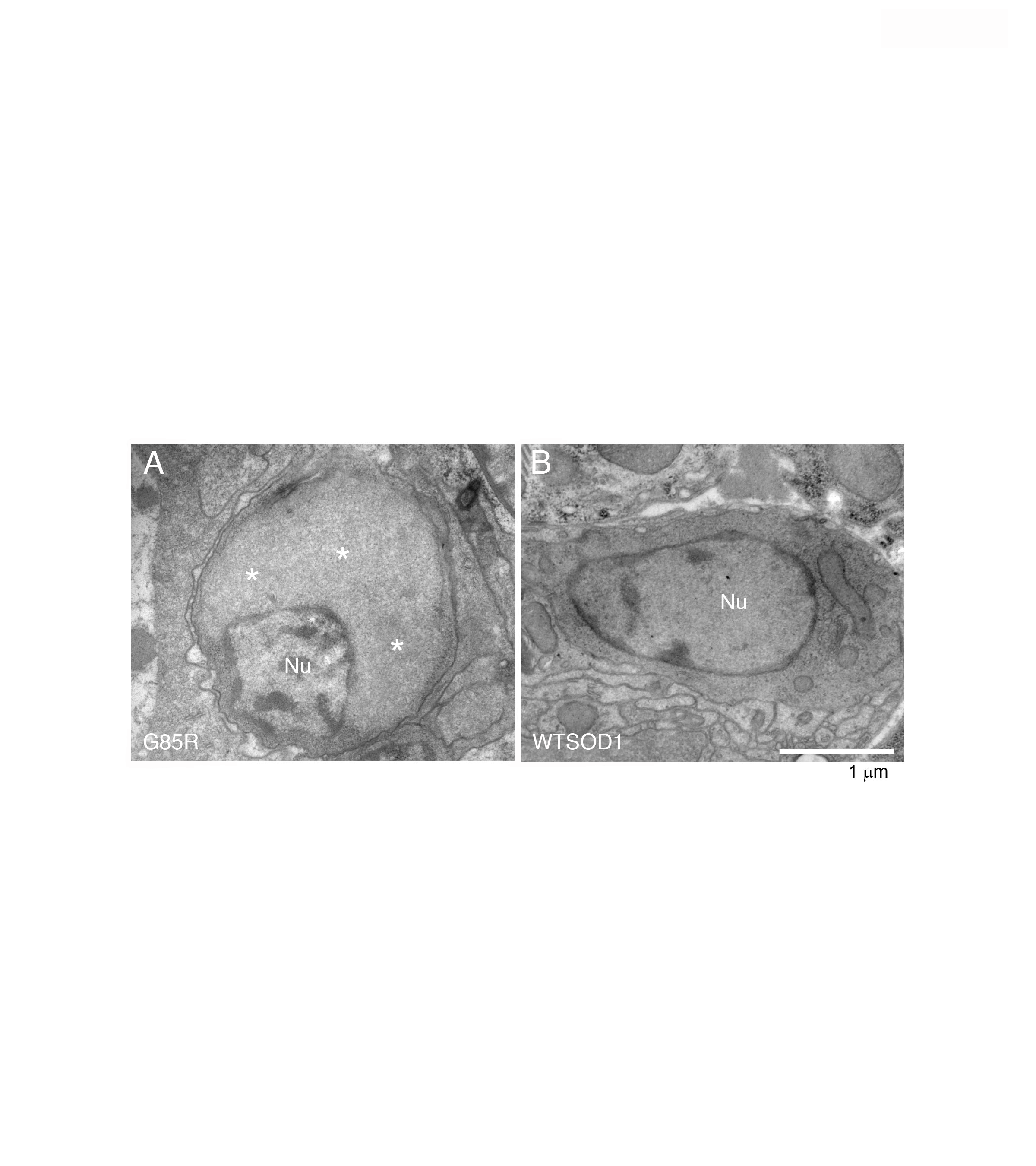

Supplement: Figure S5 — Diffuse aggregate formation in the cytosol of cell bodies of ventral nerve cord of G85R transgenic animals but not in WTSOD1 transgenics. Altered appearance of cytosol (asterisks), with “fluffy” character and with no discernible organelles in this section of a neuron cell body of a G85R animal (left), distinct from normal cytosol in cell body of a WTSOD1 transgenic animal (right). Note that the fluffy inclusion has pushed the nucleus away from the center of the cell body, similar to the position of the dense aggregate in Figure 2C. Day 4 adults were prepared by chemical immersion fixation. Scale bar 1 µm. (1.77 MB TIF) [file pgen.1000350.s005.tif]

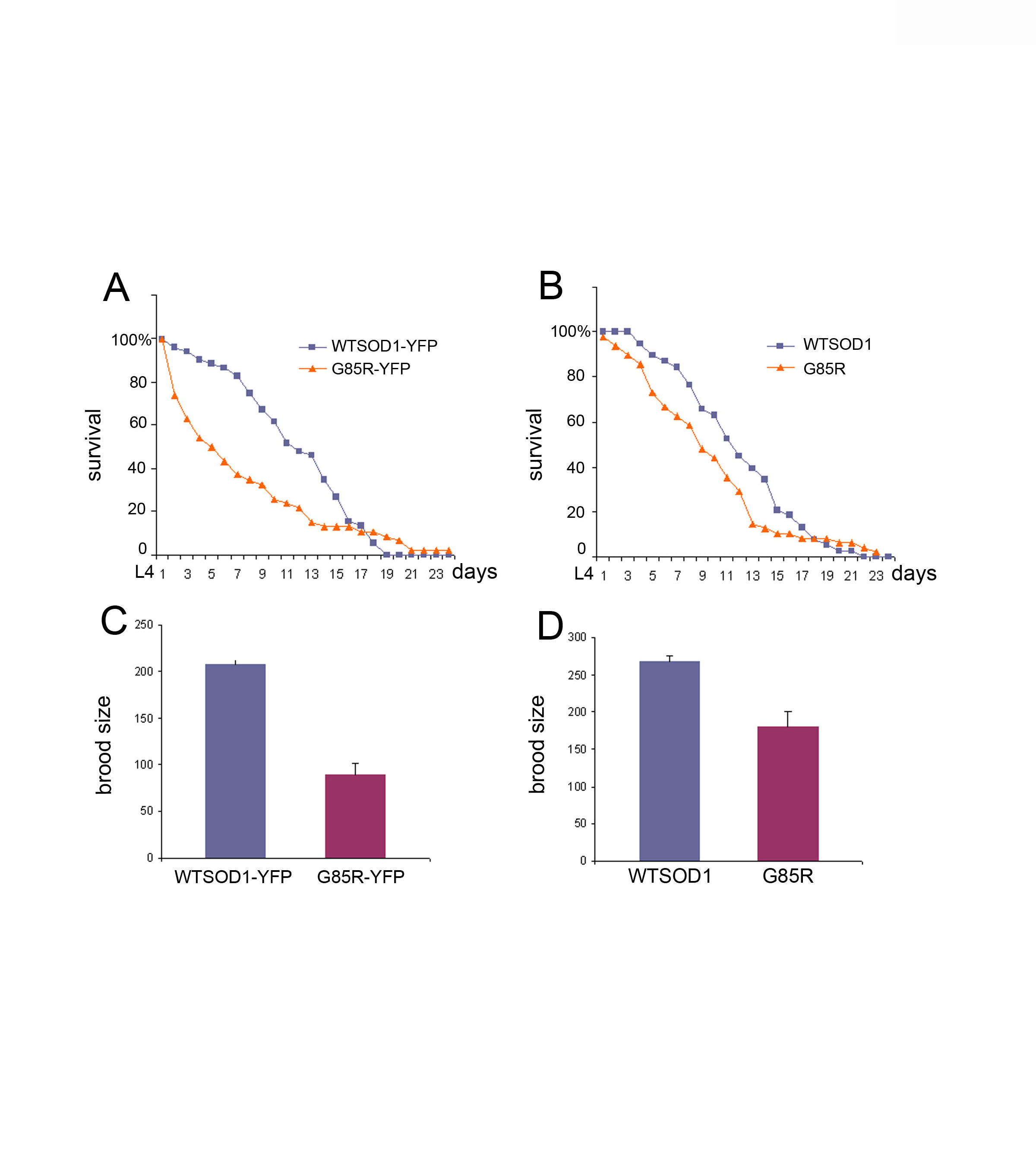

Supplement: Figure S6 — Survival curves and brood sizes of transgenic animals. A, B Survival of animals was followed from mid-L4, with death determined by failure to respond to mechanical prodding. N = 45 for each genotype. C, D Brood size was determined by counting eggs laid during the lifetime of hermaphrodite. Lines used were WTSOD1 (line 23), G85R (line 10), WTSOD1-YFP (line 51), and G85R-YFP (line 18). N = 24 for each genotype. Error bars are SEM. All animals were cultured under standard conditions at 20°C. The presence of G85R mutation affects these parameters, with a more severe effect of the G85R-YFP fusion. (0.63 MB TIF) [file pgen.1000350.s006.tif]

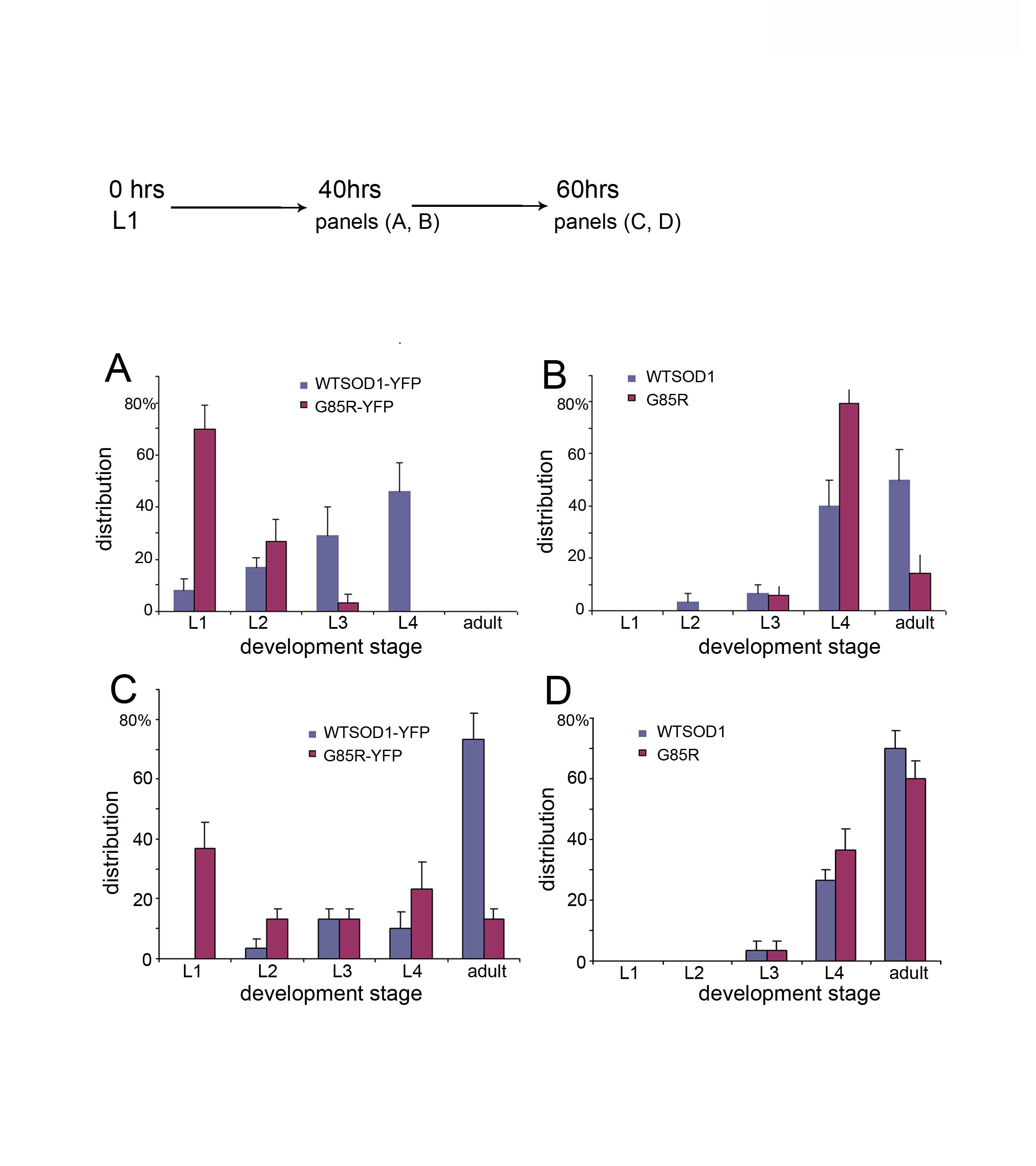

Supplement: Figure S7 — Rate of larval development of transgenic animals. Individual animals at L1 stage were selected and scored for developmental progression after 40 hr and 60 hr. G85R-YFP fusion animals were strongly delayed in development compared with G85R. N = 30 for each genotype; error bars = SEM. (0.76 MB TIF) [file pgen.1000350.s007.tif]

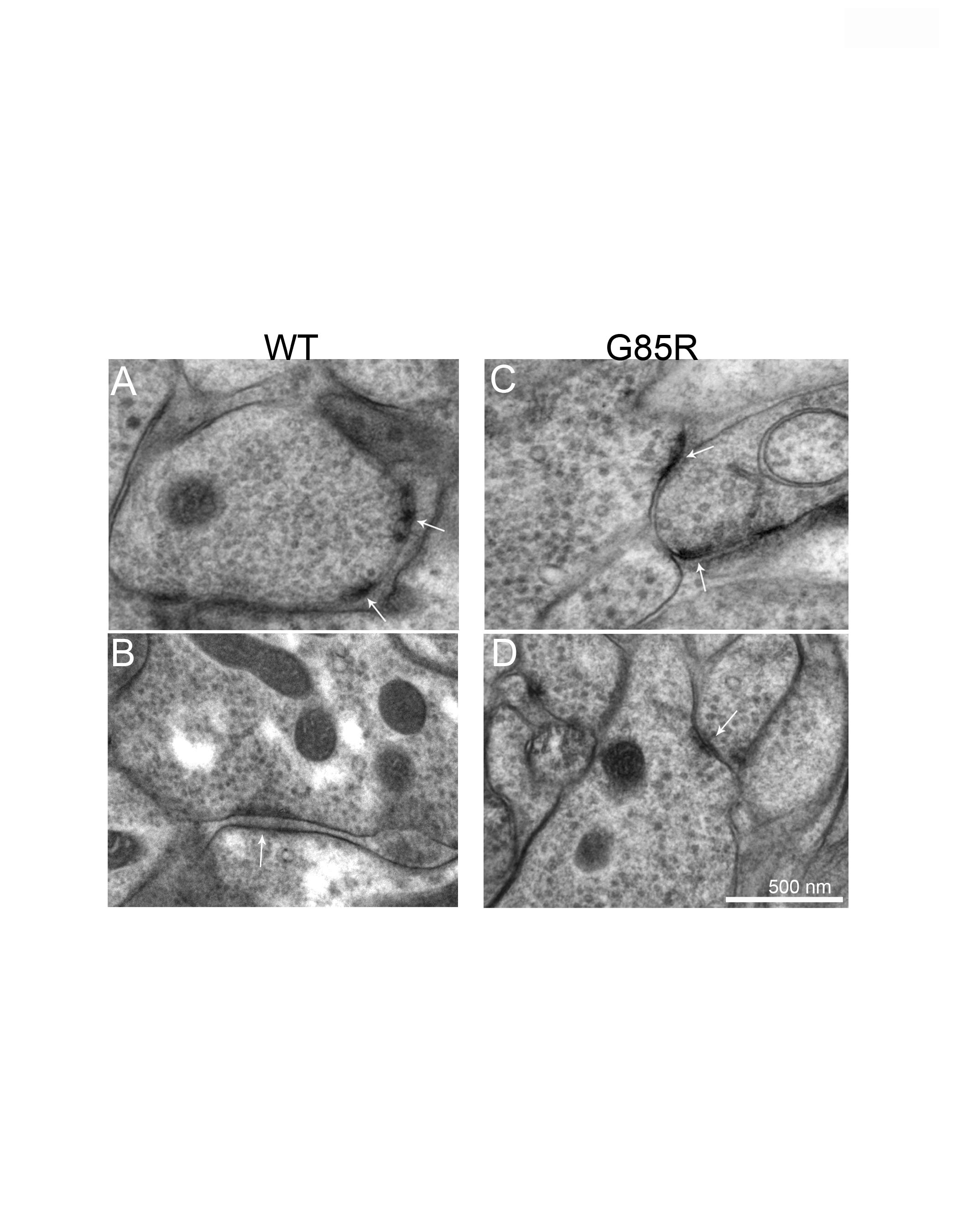

Supplement: Figure S8 — Paucity of pre-synaptic vesicles in G85R transgenic animals. Representative transverse sections of pharyngeal nerve ring from day 4 adult animals prepared by high-pressure-freezing method, with white arrows pointing to presynaptic density from the postsynaptic side. Note the paucity of presynaptic vesicles overall, and stronger depletion close to the presynaptic density in the G85R animals (panels C,D) compared with robust numbers of presynaptic vesicles in WTSOD animals (panels A,B). Scale bar, 500 nm. (1.39 MB TIF) [file pgen.1000350.s008.tif]

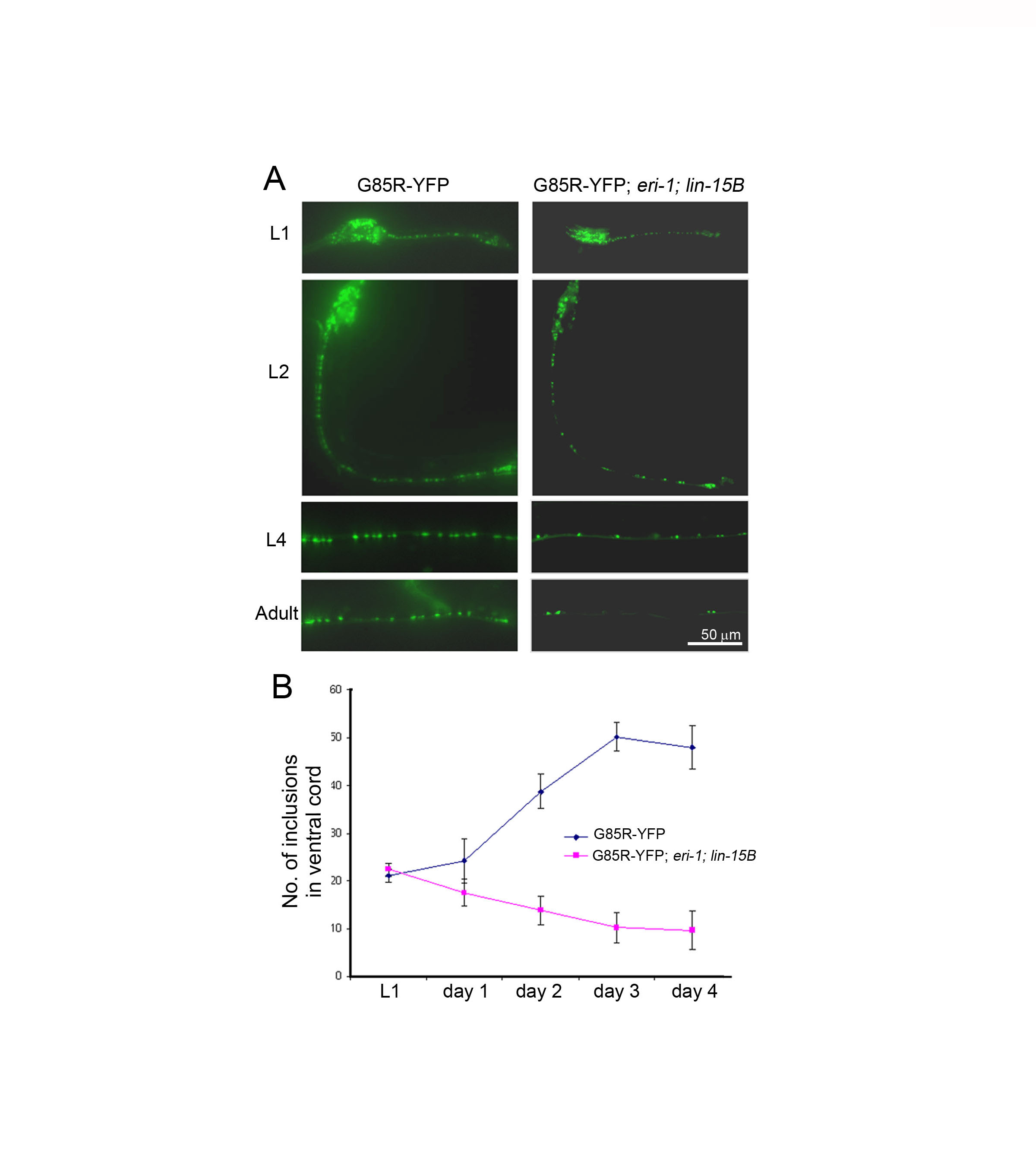

Supplement: Figure S9 — The double mutant background eri1;lin-15B that facilitates RNA interference in neurons reduces the expression/fluorescence of G85R-YFP, with corresponding reduction of the number of fluorescent inclusions. A, G85R-YFP transgenic animals of different stages with or without presence of eri-1(mg366); lin-15B(n744) were imaged. L1 and L2, whole animal views. L4 and adult, ventral cord region is shown. Bright puncta along ventral cord correspond to fluorescent inclusions in cell bodies. B, numbers of fluorescent inclusions in ventral nerve cord on successive days in the same animals. N = 18 for each genotype; error bars = SEM. (0.73 MB TIF) [file pgen.1000350.s009.tif]

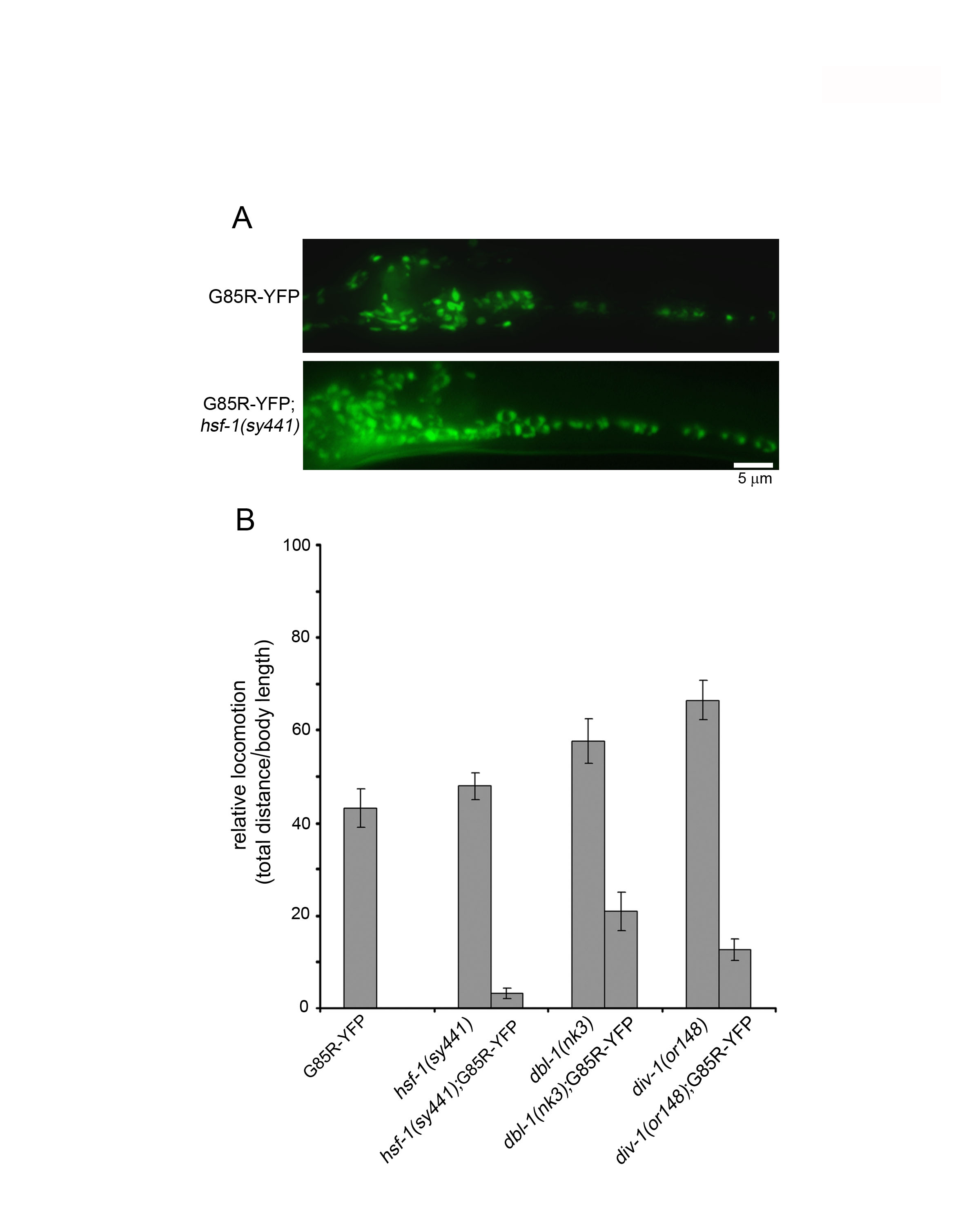

Supplement: Figure S10 — Effects of several mutant alleles on aggregation and locomotion of G85R-YFP animals. A, The hsf-1 loss-of-function allele sy441 causes an increase of aggregation. Nerve ring and anterior ventral cord of L1 stage animals are shown. B, sy441 and mutant alleles of dbl-1 and div-1 produce defective forward movement in L4 stage G85R-YFP animals. N2 locomotion (not shown) was set to 100%. (0.84 MB TIF) [file pgen.1000350.s010.tif]
