# Supplementary material for: An ALS-Linked Mutant SOD1 Produces a Locomotor Defect Associated with Aggregation and Synaptic Dysfunction When Expressed in Neurons of Caenorhabditis elegans
Source: PLoS Genet. 2009 Jan 23;5(1):e1000350. doi: 10.1371/journal.pgen.1000350 (PMC2621352; doi:10.1371/journal.pgen.1000350)
Supplement: Table S2 — Strains. (0.06 MB DOC) [file pgen.1000350.s012.doc]

Table S2. *C. elegans* strains used in the present study

| Strain | Allele | Source |
| --- | --- | --- |
|  | *nuIs152, [Punc-129::GFP-snb-1]* | (Sieburth et al., 2005) |
| KP3961 | *nuIs168(IV), [Punc-129::YFP-rab-3]* | (Simon et al., 2008) |
| KP3913 | *nuIs163(II), [Punc-129::snn-1-YFP]* | J. Kaplan |
| iY29 | *nuIs165(II), [Punc-129::unc-10-GFP]* | J. Kaplan |
| KP3948 | *lin-15B(n744);eri-1(mg366)* | (Sieburth et al., 2005) |
| PS3551 | *hsf-1(sy441)* | Caenorhabditis Genetics Center (CGC) |
| VC1157 | *C30C11.4(gk533) III/hT2[bli-4(e937) let-? (q782) qIs48](I;III)* | CGC |
| VC1348 | *dnj-19(gk649)* | CGC |
| RB1604 | *F08H9.4&srz-97(ok1976)* | CGC |
| VC562 | *rbx-1(ok782) V/nT1[qIs51] (IV;V)* | CGC |
| VC858 | *+/szT1[lon-2(e678)] I; pdi-2(gk375)/szT1 X* | CGC |
| VC212 | *fut-4(gk111)* | CGC |
| NU3 | *dbl-1(nk3)* | CGC |
| FX0903 | *dat-1(tm903)* | National Bioresource Project-Japan |
| EU548 | *div-1(or148)* | CGC |
| CB767 | *bli-3(e767)* | CGC |
| VC186 | *smo-1(ok359)/szT1[lon-2(e678)] I; +/szT1 X]* | CGC |
| LT186 | *sma-6(wk7)* | CGC |

References:

Sieburth, D., Ch'ng, Q., Dybbs, M., Tavazoie, M., Kennedy, S., Wang, D., Dupuy, D., Rual, J.-F., Hill, D.E., Vidal, M.*, et al.* (2005). Systematic analysis of genes required for synapse structure and function. Nature *436*, 510-517.

Simon, D.J., Madison, J.M., Conery, A.L., Thompson-Peer, K.L., Soskis, M., Ruvkun, G.B., Kaplan, J.M., and Kim, J.K. (2008). The MicroRNA miR-1 Regulates a MEF-2-Dependent Retrograde Signal at Neuromuscular Junctions. Cell *133*, 903-915.
